# Escaping Underground Nets: Extracellular DNases Degrade Plant Extracellular Traps and Contribute to Virulence of the Plant Pathogenic Bacterium *Ralstonia solanacearum*

**DOI:** 10.1371/journal.ppat.1005686

**Published:** 2016-06-23

**Authors:** Tuan Minh Tran, April MacIntyre, Martha Hawes, Caitilyn Allen

**Affiliations:** 1 Department of Plant Pathology, University of Wisconsin-Madison, Madison, Wisconsin, United States of America; 2 Microbiology Doctoral Training Program, University of Wisconsin-Madison, Madison, Wisconsin, United States of America; 3 Department of Soil, Water and Environmental Science, University of Arizona, Tucson, Arizona, United States of America; Michigan State University, UNITED STATES

## Abstract

Plant root border cells have been recently recognized as an important physical defense against soil-borne pathogens. Root border cells produce an extracellular matrix of protein, polysaccharide and DNA that functions like animal neutrophil extracellular traps to immobilize pathogens. Exposing pea root border cells to the root-infecting bacterial wilt pathogen *Ralstonia solanacearum* triggered release of DNA-containing extracellular traps in a flagellin-dependent manner. These traps rapidly immobilized the pathogen and killed some cells, but most of the entangled bacteria eventually escaped. The *R*. *solanacearum* genome encodes two putative extracellular DNases (exDNases) that are expressed during pathogenesis, suggesting that these exDNases contribute to bacterial virulence by enabling the bacterium to degrade and escape root border cell traps. We tested this hypothesis with *R*. *solanacearum* deletion mutants lacking one or both of these nucleases, named NucA and NucB. Functional studies with purified proteins revealed that NucA and NucB are non-specific endonucleases and that NucA is membrane-associated and cation-dependent. Single Δ*nucA* and Δ*nucB* mutants and the Δ*nucA/B* double mutant all had reduced virulence on wilt-susceptible tomato plants in a naturalistic soil-soak inoculation assay. The Δ*nucA/B* mutant was out-competed by the wild-type strain *in planta* and was less able to stunt root growth or colonize plant stems. Further, the double nuclease mutant could not escape from root border cells *in vitro* and was defective in attachment to pea roots. Taken together, these results demonstrate that extracellular DNases are novel virulence factors that help *R*. *solanacearum* successfully overcome plant defenses to infect plant roots and cause bacterial wilt disease.

## Author Summary

Plant root tips are covered by a protective sleeve of loosely attached border cells that can release a matrix containing proteins, polysaccharides, and DNA. In animal immune systems, extracellular DNA forms the backbone of neutrophil extracellular traps (NETs) deployed by immune cells to immobilize and kill invading microbes. Some animal pathogens can secrete DNases to degrade NETs and facilitate infection. We found that plant border cells release DNA-containing extracellular traps in response to the high-impact plant pathogenic bacterium *Ralstonia solanacearum*. *R*. *solanacearum* secretes two DNases that free the pathogen from these extracellular traps. The bacterium needs these DNases for full virulence and normal colonization of its host plants. This work reveals that, like animal pathogens, the plant pathogen *R*. *solanacearum* can overcome a DNA-based host defense system with secreted enzymes.

## Introduction

The growing tip of a plant root is uniquely vulnerable to infection as it moves through the dense microbial community of the soil, unprotected by cuticle or bark. However, roots are defended by tiles of loosely attached secretory cells called root border cells, which produce a matrix of proteins, polysaccharide and DNA [1,2]. It has long been known that plants deposit DNA into soil [3–6], but this extracellular DNA (exDNA) was only recently found to contribute to plant defense, possibly by trapping root pathogens [7]. For example, pea root border cells release DNA that limits root infection by the fungal pathogen *Nectria hematococca*, and treating pea root tips with DNase I accelerates necrosis caused by fungal infection [2].

ExDNA also forms the backbone of neutrophil extracellular traps, which are an important element of the animal immune system [8]. During microbial infection, neutrophils are recruited to the site of infection, where they release extracellular traps comprised of DNA matrices studded with antimicrobial compounds like histone, calprotectin, and serine proteases that can immobilize and/or kill various bacteria and fungi [8–10]. The DNA component is directly bactericidal because it chelates cations and disrupts bacterial membrane integrity [11]. NET release can be triggered by conserved molecules associated with pathogens, such as LPS, flagella, or the protein kinase C activator PMA; these are known as microbe-associated molecular patterns or MAMPs [12,13].

In response to this sophisticated physical and chemical defense, microbes have evolved ways to escape from extracellular traps. *Streptococcus pneumoniae* and *Pseudomonas aeruginosa* have modified cell surfaces that do not bind antimicrobial peptides or DNA, respectively [11,14,15]. Most commonly, pathogenic bacteria evade NETs by producing extracellular nucleases (ex DNases) that degrade the DNA backbone of the traps. Such nucleases are virulence factors for bacteria such as Group A *Streptococcus*, *Streptococcus suis*, *Staphylococcus aureus*, *Vibrio cholerae*, *Neisseria gonorrhoea* and also the eukaryotic parasite *Leishmania infantum* [16–22]. Indeed, nuclease treatment is enough to abolish the bactericidal activity of neutrophils [8]. Additionally, pathogens can convert nuclease-degraded trap components into counter-weapons that trigger neutrophil death [19].

It has been suggested that the exDNA released by plant border cells forms structures that are functionally analogous to animal NETs [23]. We will refer to these structures as NETs (Nucleic acid Extracellular Traps). Like animal pathogens, many plant pathogenic microbes secrete DNases that may help them overcome NETs. Conidiospores of the plant pathogenic fungi *Fusarium solani* and *Verticilium dahliae* release exDNases [24,25]. Bioinformatic data suggest that several plant pathogenic bacteria have nucleases with secretory signals [7]. One such pathogen, *Ralstonia solanacearum*, carries genes putatively encoding two extracellular nucleases.

*R*. *solanacearum* is a soil-borne Betaproteobacterium that causes the destructive bacterial wilt disease [26]. The pathogen has an exceptionally wide host range spanning more than 50 plant families, including economically important crops like potato, tomato, and banana, and it is notably difficult to control [26,27]. *R*. *solanacearum* is strongly attracted to root exudates by chemotaxis, and bacterial motility is required for effective root infection [28,29]. *R*. *solanacearum* enters host roots through wounds or natural openings, then multiplies and spreads rapidly in the water-transporting xylem vessels of the vascular system. The resulting mass of bacterial cells and extracellular polysaccharide obstructs water transport in the xylem and leads to wilting [30,31]. In late-stage disease, bacteria actively leave the roots and return to the soil.

Many *R*. *solanacearum* virulence factors have been identified [32], but the role of DNases in infection has not been explored. While it has been established that plant exDNA protects plants from root pathogens, only correlative evidence supports the idea that nucleases secreted by pathogens could overcome this defense. The presence of exDNA in the root border cell secretome and the putative secreted DNase genes in *R*. *solanacearum* genomes suggested that nucleases produced by this pathogen facilitate bacterial infection by digesting the exDNA in the root cap slime [7]. We directly tested this hypothesis with *R*. *solanacearum* mutants that cannot degrade exDNA. These experiments revealed that plants release extracellular traps in response to conserved pathogen factors and that *R*. *solanacearum* secretes two extracellular endonucleases that enable it to degrade and escape from these traps. Further, these enzymes contribute to root attachment, competitive fitness, plant colonization, and virulence of the pathogen.

## Results

### Plant root border cells release DNA-containing extracellular traps in response to *R*. *solanacearum*

**Fig 1:**
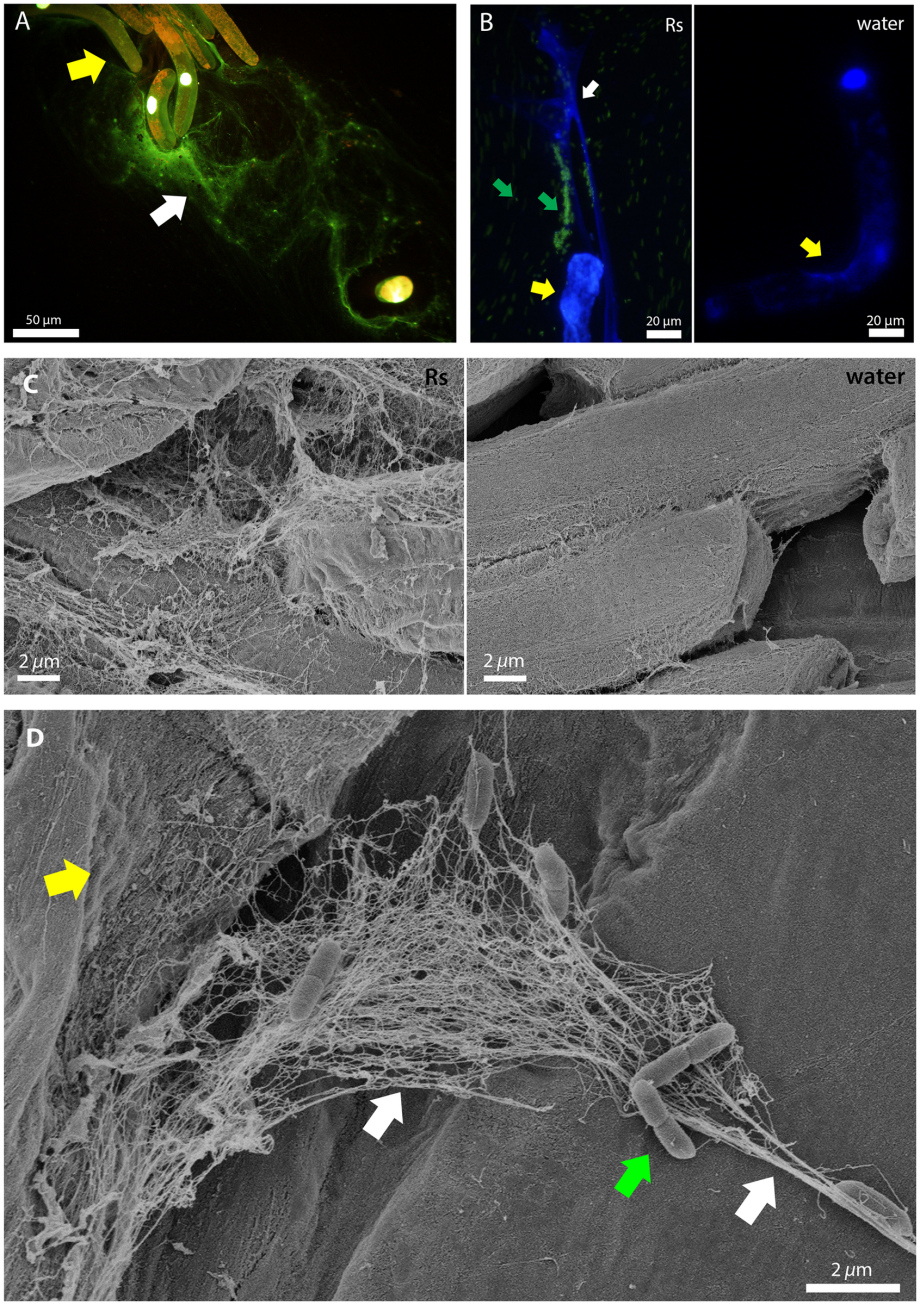
Plant root border cells release DNA-containing extracellular traps in response to *R*. *solanacearum*. (A) Fluorescence microscopy images of pea root border cells (yellow arrow) releasing extracellular DNA (white arrow, stained green or white with SYTOX Green) 30 min after exposure to *R*. *solanacearum* cells. (B) A pea root border cell (yellow arrow) treated with GFP-expressing *R*. *solanacearum* (green arrows), which are immobilized on traps containing extracellular DNA (stained blue with DAPI, white arrow). In the adjacent water control, the root border cell nuclei are stained blue but no extracellular DNA is visible. Untrapped bacteria were able to move freely in the suspension and appear blurred in the image. (C) Scanning electron microscopy showed that following treatment with *R*. *solanacearum*, pea border cells released web-like structures similar to neutrophil extracellular traps. (D) Root extracellular traps contained both small threads and thicker cables (left and right white arrows, respectively). *R*. *solanacearum* cells (green arrow) were captured by traps originating from a collapsed pea root border cell (yellow arrow).

We used pea, a model system for root border cell studies, and tomato, an economically important host of *R*. *solanacearum*, to study interactions between border cells and this pathogen. When pea border cell suspensions were inoculated with *R*. *solanacearum*, staining with DAPI and SYTOX Green revealed exDNA matrices in the suspension (Fig 1A and 1B). Some GFP-tagged bacterial cells were trapped and immobilized along these DNA strands, while non-trapped bacteria moved freely in the suspension (Fig 1B). SEM visualization of pea roots inoculated with *R*. *solanacearum* revealed web-like structures that resembled neutrophil extracellular traps. These were only present when pea roots were exposed to *R*. *solanacearum* (Fig 1C). Bacteria were trapped by NETs near border cells within an hour after inoculation and in some cases, these structures could be seen originating from collapsed border cells (Fig 1D). These NETs consisted of threads that varied in size, with the smallest about the diameter of chromatin fibers (20 nm). The bigger cables may consist of smaller threads bundled together, as has been observed in animal NETs [8].

**Fig 2:**
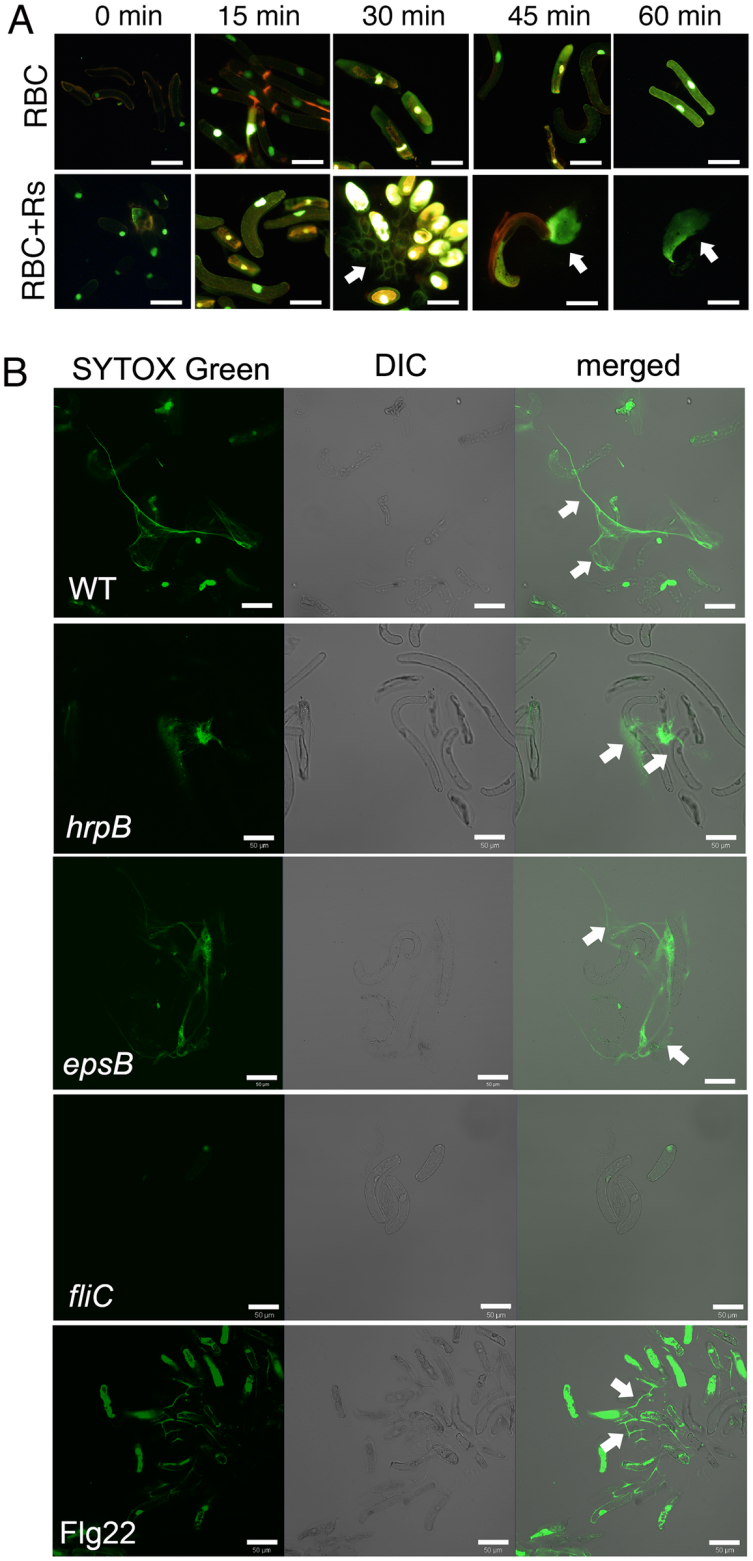
Flagella and Flg22 triggered the release of exDNA by pea border cells. (A) Kinetics of root border cell extracellular trap release. Pea root border cells (RBC) were inoculated with 10^6^ cells of *R*. *solanacearum* strain GMI1000 (Rs), the preparation was stained with SYTOX Green, and imaged over time with an epifluorescence microscope. Images are representative of two experiments and at least four images were taken for each time point. (B) Flagellin-triggered formation of border cell extracellular traps. A suspension of 10,000 pea border cells were inoculated with a 1000-fold excess (10^7^ cells) of either *R*. *solanacearum* wild-type strain GMI1000 (WT), type III secretion system mutant *hrpB*, exopolysaccharide deficient mutant *epsB*, flagellin deficient mutant *fliC*, or 20 μg/ml of flagellin-derived peptide Flg22 and stained with SYTOX Green to visualize DNA (white arrows on merged images). Live imaging was performed with a Zeiss Elyra 780 CLSM and SYTOX Green fluorescent, differential interference contrast microscopy (DIC) and merged images are shown. At least 5 images per treatment were taken from 30 min to 1 h post inoculation. The experiment was repeated three times and representative images are shown (bar = 50 μm). White arrows in (A) and (B) indicate exDNA.

Although exDNA was not associated with every border cell exposed to bacteria, it was abundant in suspensions with bacteria, while border cells mock-inoculated with water did not contain any noticeable exDNA (Fig 2A). In the water control, SYTOX Green stained only the nuclei of pea border cells. In response to *R*. *solanacearum*, border cells released DNA traps quickly. Chromatin decondensation, visible as expanding, brightly stained ovals, was visible approximately 30 min after bacteria were added, as indicated by the fact that SYTOX Green was no longer limited to the nuclei. Release of exDNA followed shortly afterwards. About 45–60 min post inoculation, many border cells collapsed and were associated with SYTOX Green-stained DNA-containing NETs (Fig 2A). Tomato border cells also released exDNA in response to *R*. *solanacearum* (S1 Fig). In contrast, non-pathogenic bacteria such as *E*. *coli*, *Sinorhizobium meliloti*, and *Pseudomonas aureofaciens* did not trigger release of exDNA from pea border cells (with the exception of *Pseudomonas fluorescens*) (S2 Fig).

### Flagella and Flg22 triggered the release of exDNA by pea border cells

Plant recognition of pathogen signals such as MAMPs or effector proteins is a critical first step in defense responses. Although previous work identified EPS as an elicitor of plant defense against *R*. *solanacearum* in resistant tomato, little is known about plant responses that occur early in the interaction [33]. To identify the pathogen signal(s) that trigger NETs release, we inoculated pea border cells with mutants of *R*. *solanacearum* missing typical plant defense elicitors such as a positive regulator of type III secretion system (*hrpB*) [34], the acidic exopolysaccharide I that is recognized by some plants (*epsB*) [33], or flagellin (*fliC*) [29]. The *hrpB* and *epsB* mutants triggered release of thread-like extracellular DNA structures indistinguishable from those induced by wild-type strain GMI1000 (Fig 2B). However, the *fliC* mutant did not induce trap formation, and border cells exposed to the *fliC* mutant were indistinguishable from those treated with water alone, even though *R*. *solanacearum fliC* cells were in close proximity to pea border cells (S3 Fig). Treating border cells with 20 μg/ml of the conserved flagellin-derived peptide Flg22 also triggered some trap release. These results suggest that extracellular trapping is a previously undescribed element of MAMP-triggered immunity.

### Histones and DNA contribute to the bactericidal activity of pea root border cells on *R*. *solanacearum*

**Fig 3:**
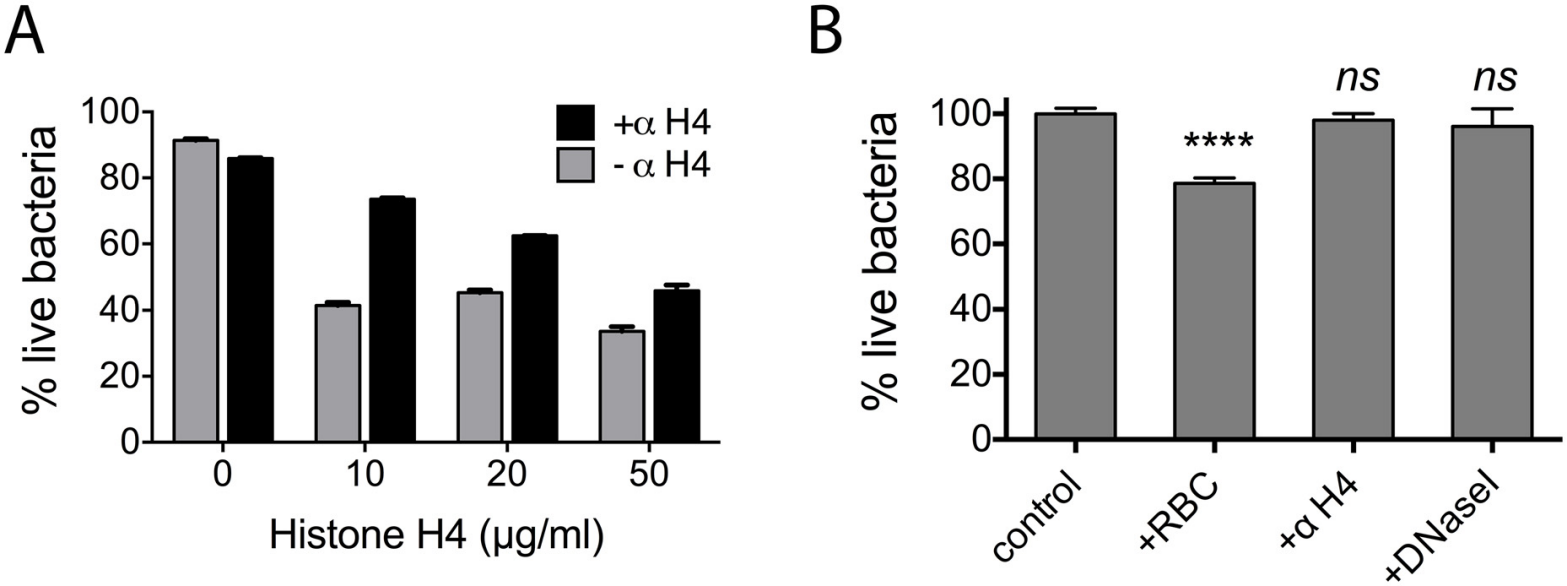
Histones and DNA contribute to the bactericidal activity of pea root border cells. (A) Histone H4, a component of the root cap secretome, is bactericidal to *R*. *solanacearum*. Percent live bacteria in the presence of histone H4 was determined by the BacLight LIVE/DEAD staining kit. A standard curve of percentage of live cells relative to SYTO9/PI fluorescence intensity was constructed using known ratios of live/dead bacteria (R^2^ = 0.99). The experiment was repeated twice, each with three technical replicates. (B) Bactericidal activity of root border cells on *R*. *solanacearum*. This effect was blocked by addition of either anti-Histone H4 antibody or DNase I. Asterisks indicate treatment significantly different from the bacteria-only control (one-way ANOVA, **** p<0.0001). Bars represent the mean of at least three independent experiments, each comprised of six technical replicates.

The bactericidal activity of animal NETs is partly the result of histone proteins, which disrupt charges on bacterial surfaces [35]. Interestingly, histone H4 is also found in the plant root cap secretome [36]. Microscopic studies showed that *R*. *solanacearum* cells were immobilized in exDNA traps, but it was unclear if the traps were toxic to the pathogen. To test the hypothesis that plant histones contribute to the bactericidal effect of border cells, we first measured *R*. *solanacearum* survival following exposure to human histone H4, which is 97% identical to plant histone H4 (NIH Histone Sequence Database). At 10 μg/ml, histone H4 killed about 50% of the bacteria in 3 h, as determined by Live/Dead staining. Treatment with anti-Histone H4 antibody partially reversed the bactericidal effect of histone H4 on *R*. *solanacearum* (Fig 3A).

To determine if border cell trapping can kill *R*. *solanacearum*, we measured bacterial survival following 3 h incubation with pea border cells. About 25% of the *R*. *solanacearum* population was killed compared to a water-treated control population. Freeing the bacteria from the traps prevented bacterial killing, because when the suspension was treated with DNase I there was no difference in survival between bacterial populations exposed to border cells and a water-treated control population (Fig 3B). Adding anti-Histone H4 antibody to the challenging pea border cells also fully protected the pathogen, possibly because the antibody either prevented bacteria from binding to histones in exDNA traps or neutralized the antimicrobial activity of histone H4. This suggests that both exDNA and histone H4 are required for effective killing of *R*. *solanacearum* by border cell traps.

### The *R*. *solanacearum* genome encodes two functional extracellular DNases

The genome of *R*. *solanacearum* strain GMI1000 contains two putative endonuclease genes, *nucA* (locus tag: RSc0744) and *nucB* (locus tag: RSc2452), and both genes encode predicted secretion signals (Signal P 4.0) (S4 Fig). Expression levels of *nucA* and *nucB* are high in two different *R*. *solanacearum* strains growing in rich medium and during tomato pathogenesis [37]. Both *nucA* and *nucB* belong to the core *Ralstonia solanacearum* genome and are highly conserved in all 28 sequenced *R*. *solanacearum* strains in the MaGE RalstoniaScope database and in the opportunistic human pathogen *R*. *pickettii*, but are not present in the related free-living bacterium *R*. *eutropha* (http://www.genoscope.cns.fr/agc/microscope/home/index.php) [38].

**Fig 4:**
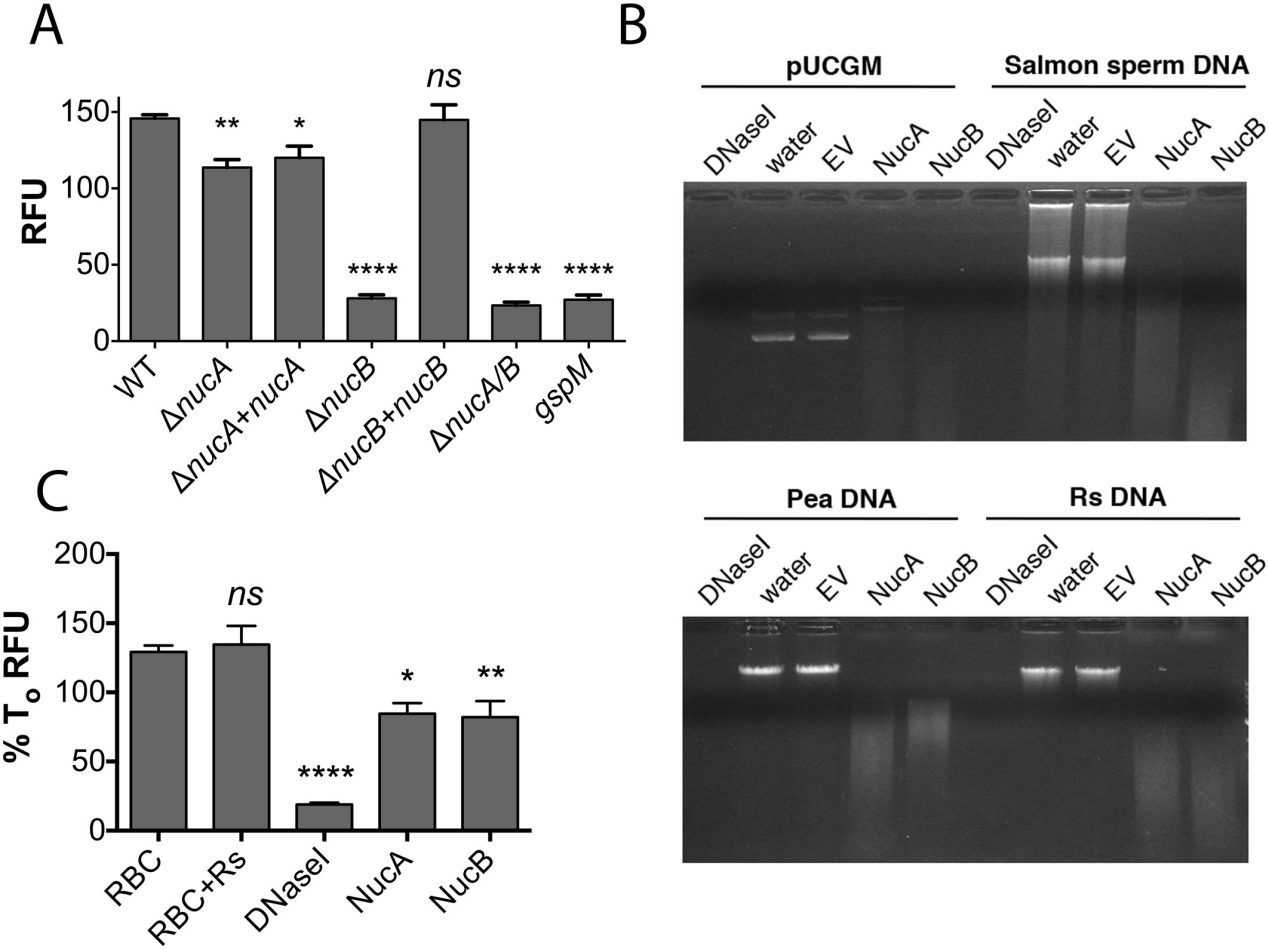
*R*. *solanacearum* genes encode two secreted DNases. (A) DNase activity of cell-free bacterial culture supernatant from *R*. *solanacearum* strains grown in minimal medium, measured by DNase Alert assay on a fluorescence plate reader at 37°C over 3 h. Bars represent mean relative fluorescence units normalized to A_600_ of overnight culture. (B) Activity of purified nucleases on different DNA substrates. 1 μg of purified NucA or NucB were incubated with 1 μg of each DNA substrate: *R*. *solanacearum* genomic DNA, pea DNA, supercoiled plasmid DNA (pUCGM) and salmon sperm DNA) for 30 min at 37°C. Results were analyzed by electrophoresis in a 1% agarose gel. (C) NucA and NucB degrade DNA from root border cell traps. Pea border cells were incubated with *R*. *solanacearum* in the presence of NucA, NucB or DNase I as control. Relative DNA amount was measured by SYTOX Green fluorescence after 6 h of incubation. Asterisks indicate differences from the wild-type (one-way ANOVA, *P<0.05, **P<0.01, ****P<0.0001). Abbreviations: WT, wild-type strain; *ΔnucA*, *R*. *solanacearum nucA* mutant; *ΔnucB*, *R*. *solanacearum nucB* mutant; *ΔnucA/B*, *R*. *solanacearum* double nuclease mutant; nucBcom or nucBcom; *R*. *solanacearum* mutant complemented with the corresponding wild-type gene; RBC, root border cells; Rs, *R*. *solanacearum* cells.

To determine the biological role(s) of *R*. *solanacearum’s* extracellular nucleases, we constructed deletion mutants lacking *nucA*, *nucB*, or both *nucA* and *nucB* (referred to hereafter as *ΔnucA/B*) (S4 Fig). All three DNase mutants produced significantly less exDNase activity than the parental strain (Fig 4A). The *ΔnucA* mutant showed a small reduction in exDNase activity *in vitro*, but the Δ*nucB* and *ΔnucA/B* double mutants produced only about 18% of wildtype DNase activity levels (in relative fluorescence units), indicating that NucB is responsible for most of the exDNase activity in strain GMI1000 Adding a copy of *nucB* to the *ΔnucB* mutant restored wild-type nuclease activity levels. Complementation restored the wild-type phenotype to *ΔnucB* and partially restored *ΔnucA* mutant. The individual and double nuclease mutants did not express the corresponding genes, and the complemented strains had transcript levels comparable to those of wild-type strain GMI1000 (S4B and S4C Fig).

Bacterial culture supernatants contained nuclease activity, indicating that NucA, NucB or both enzymes may be secreted. The Type II secretion system (T2SS) exports many cell wall degrading enzymes in *R*. *solanacearum* [39]. Cell-free culture supernatants of a GMI1000 *gspM* mutant, which lacks an essential component of the T2SS, had only 18% of wildtype DNase activity, similar to the activity produced by the *ΔnucB* mutant. This suggests that *R*. *solanacearum* exports at least NucB via the T2SS.

Wild-type strain GMI1000 grew on minimal medium supplemented with salmon sperm DNA, but growth was slow and weak, reaching log phase after approximately 24 h and stationary phase after 60 h. The *ΔnucA/B* mutant grew even less (S5 Fig). These results indicate that *R*. *solanacearum* needs exDNases to use exDNA as a carbon source, but DNA is not an ideal carbon source for this organism *in vitro*.

### NucA and NucB are non-specific DNases with different cation preferences

Bioinformatic analysis suggested that NucA and NucB are distinct DNases (S3 Fig). Purified NucA and NucB were approximately 30 kDa and 15 kDa, respectively, in agreement with bioinformatic predictions (S6A Fig). We could purify a soluble form of NucA from *E*. *coli* only by removing the gene region encoding the 55 N-terminal residues containing a predicted transmembrane domain; this supports the bioinformatic prediction that NucA localizes on a bacterial membrane. To confirm that NucA is secreted through the inner membrane, we used a NucA::PhoA fusion construct and an alkaline phosphatase plate assay. Colonies of *E*. *coli phoA* ^-^ strain KS272 expressing the NucA-PhoA fusion were bright blue, similar to those of the FtsI::PhoA positive control (S6B Fig), indicating that the NucA catalytic domain is located in the periplasm or outside the cell. In addition, purified NucA and NucB digested all DNA substrates tested, including salmon sperm DNA, *R*. *solanacearum* genomic DNA, pea genomic DNA and supercoiled plasmid DNA, indicating that they are non-specific endonucleases (Fig 4B). NucA required Mg for full activity, but NucB was fully active without a cationic cofactor (S6C and S6D Fig).

### Purified NucA and NucB degrades exDNA from pea border cells and releases trapped *R*. *solanacearum*

To determine the effects of purified DNase on root border cell NETs, we measured total DNA from root border cells inoculated with *R*. *solanacearum* using SYTOX Green (Fig 4C). Addition of 10 units of commercial DNase I reduced SYTOX Green fluorescence approximately 6-fold. Adding 2 μg of purified overexpressed NucA or NucB also reduced relative fluorescence to 30% of the no enzyme control, demonstrating that *R*. *solanacearum* NucA and NucB can degrade the DNA of plant border cell traps. The total fluorescence signal of pea border cells was unchanged 6 h after addition of *R*. *solanacearum* cells, suggesting that no *de novo* DNA synthesis occurred in response to *R*. *solanacearum*.

For a more dynamic perspective on the effects of nucleases on trapped bacteria, we filmed *R*. *solanacearum* interactions with pea border cells using light microscopy. Wild-type bacteria, which were initially highly motile, were immobilized shortly after they were added to a border cell suspension (S1 Video). However, the wild-type cells were able to escape from traps after 1 h and they remained free at 24 h (S2 Video). In contrast, the Δ*nucA/B* double nuclease mutant was still immobilized in NETs 24 h after incubation with pea border cells (S3 Video). Treatments with purified NucA (S4 Video) or NucB (S5 Video) released the trapped Δ*nucA/B* bacteria. Together, these data indicate that *R*. *solanacearum* cells immobilized by plant NETs can be freed by either their endogenous exDNases or exogenously applied nucleases.

### *R*. *solanacearum* needs exDNases for virulence on tomato

**Fig 5:**
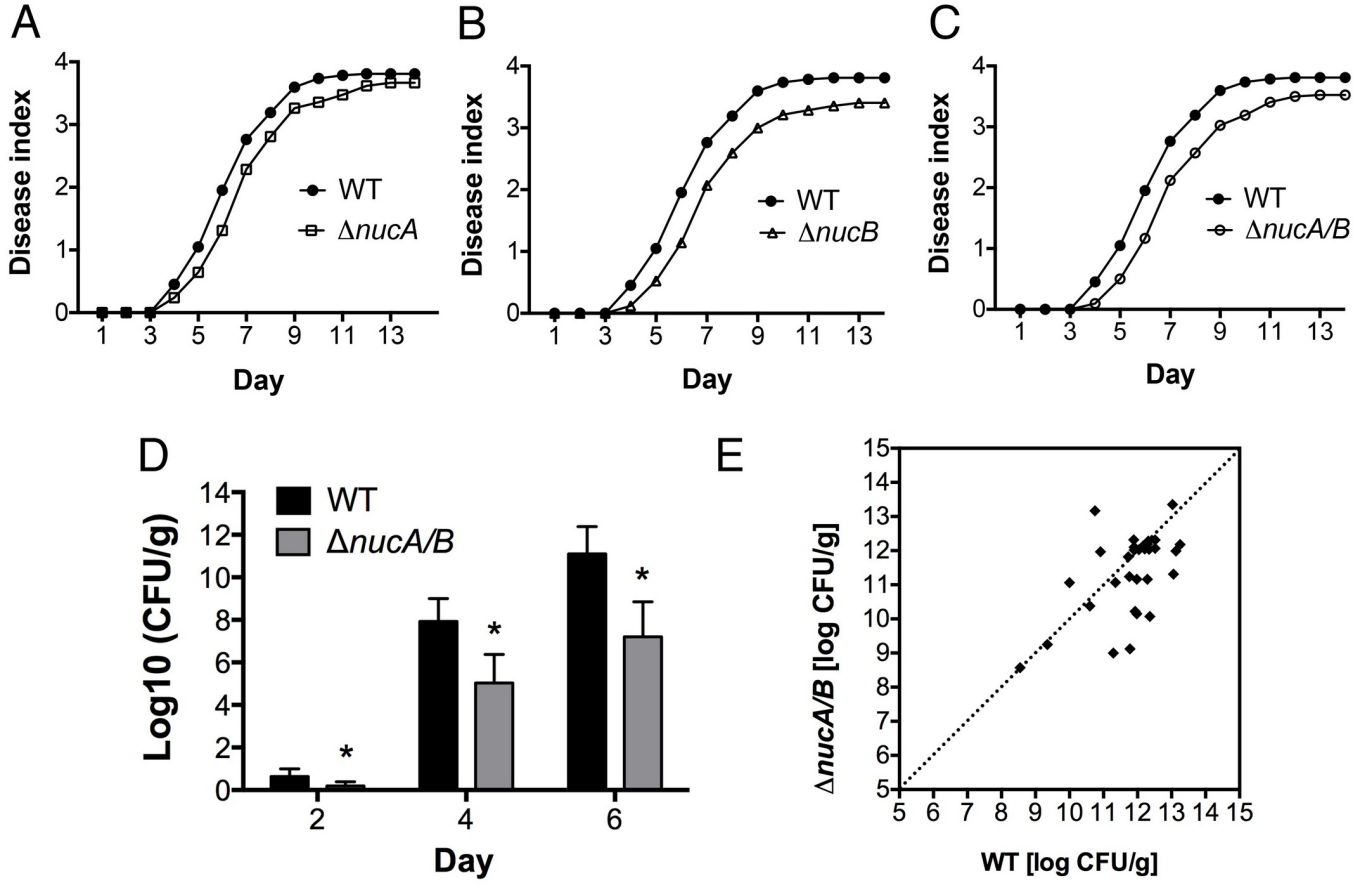
*Ralstonia solanacearum* needs exDNases for full virulence on tomato. (A-C) Disease progress on tomato. Twenty-one-day-old wilt-susceptible tomato plants (cv. Bonny Best) were inoculated by soil-drenching with 1x10^7^ CFU/g soil of *R*. *solanacearum* wildtype strain GMI1000, *ΔnucA*, *ΔnucB*, and the *ΔnucA/B* double nuclease mutant. Plants were rated daily using a 0–4 disease index scale (0: no leaves wilted, 4: all leaves wilted). Each point represents the average disease index of 42 plants from three independent experiments. The virulence of all three nuclease mutants was different from wild-type strain (repeated-measures ANOVA, *P*<0.05). (D) The *R*. *solanacearum ΔnucA/B* double nuclease mutant was impaired in plant colonization, measured as bacterial population size in mid-stems of susceptible tomato (cv. Bonny Best) inoculated by soil-drenching with 10^7^ CFU/g soil of either GMI1000 WT or the *ΔnucA/B* mutant. Data were obtained by grinding and serial dilution plating a mid-stem section at first sight of symptoms. Results are representative of two independent experiments with at least 10 plants per treatment (Student’s t-test, *P*<0.05). (E) The *R*. *solanacearum ΔnucA/B* nuclease double mutant had lower competitive fitness in tomato plants than the wildtype strain. Tomato plants were soil-drenched with a 1:1 mixture containing 1x10^8^ CFU/ml each of GMI1000-gfp and *ΔnucA/B*. When wilt symptoms first appeared on a plant, the population size of each strain was determined by grinding a mid-stem slice and serially dilution plating on both tetracycline and kanamycin+gentamycin CPG plates. Data are representative of two independent experiments each including at least 30 plants. Competitive Index of *ΔnucA/B*/ WT = 0.88 (Wilcoxon signed-ranked test, *P*<0.05).

We used the nuclease-deficient mutants to test the hypothesis that exDNases contribute to *R*. *solanacearum* bacterial wilt virulence at early stages of infection by helping the pathogen escape from the exDNA traps produced by plant roots. Susceptible tomato plants were inoculated with the wild-type strain or nuclease mutants using a soil-drenching method that introduces bacteria to the soil near unwounded plants. This requires the pathogen to follow its natural route of infection through an intact host root system. All three exDNase mutants were quantitatively reduced in virulence compared to the parental strain (*P*<0.05, repeated measures ANOVA) (Fig 5A–5C).

The delayed disease development that we observed in tomato plants inoculated with Δ*nucA/B* could be the result of reduced plant colonization ability. To evaluate this hypothesis, we quantified bacterial populations in the mid-stems of wilt-susceptible tomato plants following soil-drench inoculation with either wild type or the Δ*nucA/B* mutant. The population sizes of the wild-type strain were at least two orders of magnitude larger than those of Δ*nucA/B* at 4 and 6 days post inoculation (Fig 5D) (*P*<0.05, Student’s t-test).

To measure the ability of a nuclease-deficient strain to compete effectively with its wild-type parent in a plant host, we inoculated tomato plants with a 1:1 mixture of wild-type and *ΔnucA/B* mutant cells and measured the population size of each strain in tomato mid-stems when wilt symptoms first appeared. The wild-type bacterium slightly outcompeted the Δ*nucA/B* mutant with a competitive index of 0.88 (Fig 5E; *P*<0.05, Wilcoxon signed-ranked test).

### *R*. *solanacearum* exDNases contribute to root stunting and bacterial attachment to pea roots

**Fig 6:**
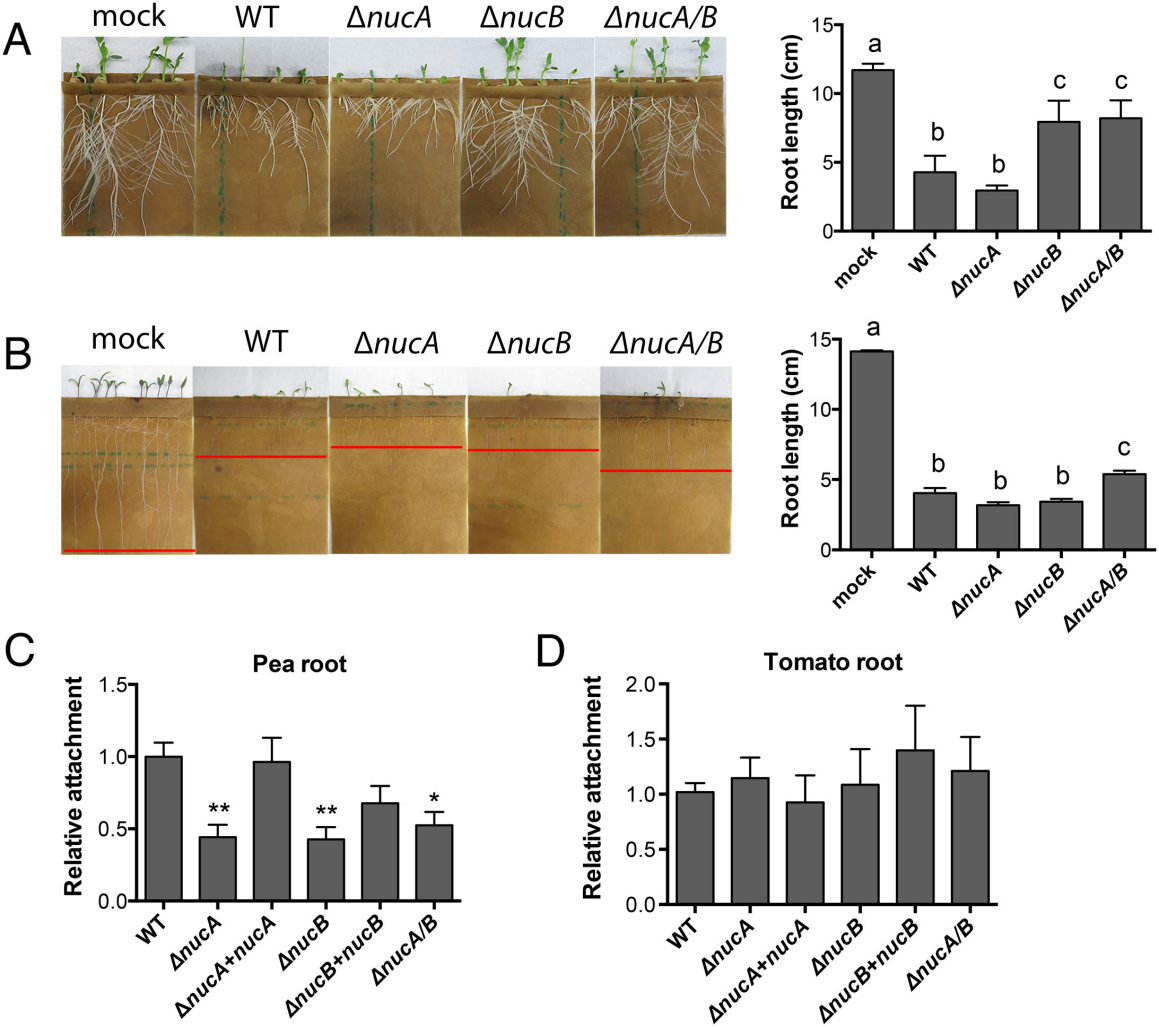
*R*. *solanacearum* exDNases contribute to tomato and pea root stunting and bacterial attachment to pea roots. Seedlings of (A) pea (cv. Little Marvel) and (B) tomato (cv. Bonny Best) were grown on water agar for 2–3 days, inoculated with 10^7^ cells (pea) or 10^6^ cells (tomato) of *R*. *solanacearum* wild-type strain GMI1000 or exDNase mutant strains as indicated and grown in germination pouches at 24°C (pea) or 28°C (tomato) for 10 days. Red bars indicate approximate root tip length. Representative results of two independent experiments are shown. Bar graphs show average length of tomato root determined using measurements with ImageJ. Bars that have the same letter are not significantly different (Tukey’s HSD test, *P*<0.01). (C) and (D) ExDNase mutants are defective in attachment to pea roots, but not to tomato roots. Three-day old axenic tomato or pea seedlings were incubated for 2 h with 2.5x10^3^ CFU of either wild-type strain GMI1000 or the *ΔnucA/B* mutant. Excised roots were washed in sterile water and blotted dry. Four roots were pooled as a technical replicate, ground, and serially dilution plated to quantify attached bacteria. Data shown are from three independent experiments; each contained 5 technical replicates. Bars represent proportion of wild-type root attachment (one-way ANOVA, *P< 0.05, ** *P*< 0.01).

To evaluate the contribution of bacterial exDNases to bacterial virulence on plant roots, we inoculated axenic tomato and pea seedlings in germination pouches with *R*. *solanacearum*. Ten days after inoculation, mock-inoculated pea and tomato plants developed healthy root systems, but roots of plants inoculated with wild-type *R*. *solanacearum* were severely stunted (Fig 6A and 6B). Pea roots inoculated with the Δ*nucA* mutant were stunted like those inoculated with the wild-type strain, but peas inoculated with either Δ*nucB* or Δ*nucA/B* grew nearly as well as the mock-inoculated control plants (Fig 6A). On tomato seedlings, only the *ΔnucA/B* double nuclease mutant caused significantly less root stunting than wild-type (Fig 6B), suggesting that both nucleases additively contribute to symptom development on this host.

Attachment to root surfaces is important for *R*. *solanacearum* infection [40]. If exDNA in the root cap trap matrix blocks pathogen access to the root surface, we would expect that a *R*. *solanacearum* strain lacking exDNases would not attach to plant roots as well as the wild type strain. We used a seedling root attachment assay to test the hypothesis that the reduced virulence of nuclease mutants in the soil-drenching assay was caused by poor root attachment. There were no significant differences among the strains in attachment to tomato seedling roots, although the *ΔnucB* complemented strain trended towards better attachment. In contrast, the nuclease mutants did not attach to pea roots as well as the wild-type strain, and complementation restored wild-type levels of attachment to pea roots (Fig 6C and 6D). This indicates that exDNases can play a critical role in *R*. *solanacearum* attachment to seedling root surfaces, at least on pea.

## Discussion

Very soon after the discovery that animal neutrophil cells immobilize and kill pathogens with DNA-containing extracellular traps, it was found that that microbes escape from these traps by means of secreted nucleases [8,15]. A few years later Hawes and colleagues showed that plant root border cells, which are quite biologically distinct from the neutrophils of the animal circulating immune system, also release DNA-containing extracellular traps that protect plants from pathogens [2]. Because this effective defense likely put strong selection pressure on pathogen populations, and because enzymatic degradation is a straightforward counter-defense, we hypothesized that the root-infecting plant pathogen *R*. *solanacearum* also uses nucleases to escape from DNA-containing traps.

NET release appears to be a specific response to pathogen recognition. Consistent with a previous report that *E*. *coli* was not trapped by maize border cells [42], we found that pea border cells did not release NETs in response to the non-pathogenic bacteria *P*. *aureofaciens*, *S*. *meliloti*, or *E*. *coli*. Similarly, non-pathogenic fungi were not aggregated or inhibited by pea roots and a fungal pathogen was not inhibited by non-host plant species [43]. These observations suggest that plant NET release is triggered by a specific signal.

Border-like cells of flax and *Arabidopsis* respond to several MAMPs, including peptidoglycan and Flg22, with ROS production and other innate defenses collectively known as MAMP-triggered immunity or PTI [44]. Bacterial flagella are potent inducers of PTI; flagellin-mediated responses account for an estimated 90% of PTI in tomato [45]. We found that the *Pseudomonas*-derived flagellin sub-peptide Flg22 could trigger NET production by itself. Our finding that border cells recognize this well-known MAMP suggests that NET trapping is an element of PTI [46]. Consistent with this idea, an aflagellate *fliC* mutant of *R*. *solanacearum* did not induce NET release. We previously found that *R*. *solanacearum* flagellin is not recognized by the *Arabidopsis* FLS2 receptor, probably because the N-terminal *R*. *solanacearum* flagellin sequence that corresponds to Flg22 contains multiple polymorphisms compared to the canonical *Pseudomonas* flagellin Flg22 sequence [47]. Therefore, the pea root border cells in our experiment recognized both the synthetic Flg22 peptide and an element of *R*. *solanacearum* flagellin that is different from Flg22. This additional MAMP could be the recently-discovered FlgII-28 peptide, which is recognized by the tomato FLS3 receptor [48].

It is not clear why NETs were not triggered by the flagella of non-pathogenic bacteria. *E*. *coli* DH5α may not have triggered NET release because *E*. *coli* lab strains are often poorly flagellated [49]. Some non-pathogenic rhizosphere-dwelling microbes like *P*. *fluorescens* and *S*. *meliloti* have developed mechanisms to suppress plant NET release. Additional studies are needed to explore this interesting result.

Flagellin can trigger NET release in animal neutrophils [50], as can calcium [51], LPS [8] or nitric oxide (NO) [52]. Interestingly, NO is also a plant signal molecule that connects multiple defense pathways [53]. Several lines of evidence indicate that ROS leads to animal cell NET release by activating the release and translocation of neutrophil elastase and myeloperoxidase into the nucleus and promoting chromatin decondensation [54–56]. Phorbol-12-myristate-13-acetate (PMA), a widely-used animal NET activator, also activates protein kinase C-like proteins in maize plants [57]. Since plant and animal defense responses are activated via a parallel series of MAPK cascades, with several conserved MAMP receptors [58], we speculate that similar plant signaling pathways may govern the formation of border cell extracellular traps, although the exact molecular machinery that orchestrates plant NET production still requires further study.

We found that histone H4, the only histone detected in root cap mucilage [1], is bactericidal to *R*. *solanacearum* cells *in vitro*, and treatment with anti-H4 antibody prevented pea border cells from killing the pathogen. Histones are toxic to a wide range of bacteria, possibly because they bind to and disrupt bacterial membranes [59]. Our results suggested that histone H4 can kill plant pathogens, either by direct contact and/or by binding bacterial cells to DNA strands where they are exposed to concentrated antimicrobial compounds. In addition to histones, root border cells have a distinct metabolic profile that is highly enriched in secondary metabolites like flavonoids and triterpene saponins that could kill or inhibit soil pathogens that contact NETs [60].

It is well established that root border cells contribute to plant health [61]. Although every plant species produces a fixed range of border cells each day [62], border cell production is also cultivar-dependent [41]. Intriguingly, we observed that seedlings of a bacterial wilt-resistant tomato breeding line, Hawaii 7996, released almost three times as many border cells as a wilt-susceptible tomato cultivar, Bonny Best (S7A Fig). Further, border cells from the resistant line appeared to be surrounded by a thicker mucilage layer (S7B Fig). Together with our finding that root border cells trap and exclude the bacterial wilt pathogen, this preliminary observation suggests the testable hypothesis that more abundant and more mucoid border cells increase plant resistance to soil-borne diseases. If additional studies find a consistent correlation between number of root border cells and wilt resistance, plant breeders seeking to reduce crop losses to root-infecting pathogens might be able to select for this easily quantifiable trait.

Wild-type *R*. *solanacearum cells* quickly escaped from pea root border cell NETs, but a mutant missing both nucleases remained immobilized. Adding purified nucleases (NucA, NucB or DNase I) reversed bacterial trapping. Together these two findings are direct evidence that, like many animal pathogenic microbes, this plant pathogen uses nucleases to escape extracellular DNA traps from host root border cells. In addition, we demonstrated that exDNases are required for full bacterial wilt virulence. Interestingly, although the disease progress curves of the three mutants differed from those of wild type, the curves of the three mutants were not significantly different from each other. This suggests that although loss of either or both exDNases reduced virulence, some mutant cells escaped or avoided the traps at this relatively high inoculum density. It appears that these escaped cells could then infect normally, although at a reduced rate. To the best of our knowledge, this is the first report that DNase contributes to plant pathogenesis. We suspect this will prove to be a widespread counter-defense mechanism since extracellular DNases are produced by many fungal and bacterial phytopathogens [25,63–65].

Interestingly, the ability of *R*. *solanacearum* to escape from NETs correlated with its ability to attach to and inhibit pea root development. Although pea is not a natural host of *R*. *solanacearum* [66], in our assay conditions it caused necrosis and stunting on pea root and inhibited growth of the aboveground plant parts. These symptoms did not occur on pea seedlings inoculated with the double nuclease mutant. We also found that nucleases were not required for attachment to roots of tomato, a natural host plant. This difference could be due to the smaller number of border cells released by tomato seedlings. In contrast to pea roots, which make thousands of root border cells per day, tomato roots produced only approximately 200 border cells daily (S7 Fig). Therefore, tomato border cells may not trap *R*. *solanacearum* cells very effectively at the seedling stage when plants were collected for the pouch assay. In larger plants, lateral root development with more root tips likely results in more border cells; also, border cells are renewed daily as roots move through the soil [61]. As a consequence, border cell NETs could efficiently limit bacterial entry to whole plants growing in soil, as observed in our plant virulence assays.

Both of the *R*. *solanacearum* extracellular nucleases contributed to stunting symptoms in tomato roots, but NucB was more important in pea root infection than NucA. This may be because NucA requires a cation cofactor and magnesium availability may differ between pea and tomato roots. In addition, exDNA can sequester cations from bacterial cell surfaces [11]. If the abundant pea root border cells release large amounts of exDNA, the resulting cation sequestration could inhibit NucA. Differential inhibition of these enzymes by natural DNase inhibitors like actin [67], which is present in the pea root cap secretome [1], could also affect the relative activity of each DNase in the rhizosphere.

The double nuclease mutant grew almost as well as the wild-type strain in a paired-strain competition plant assay intended to detect quantitative differences in strain fitness [68]. This result may appear inconsistent given the significantly lower colonization ability and virulence of the Δ*nucA/B* double mutant. However, we speculate that when the two strains were inoculated together, nucleases secreted by the wild-type strain functionally complemented the Δ*nucA/B* mutation This would allow the double mutant to escape NETs and attach to roots, and colonize stems almost as well as its wild-type parent.

DNA is rich in carbon, nitrogen and phosphorus and is abundant in soil and marine sediments [70,71]. Microbial nucleases can thus be important for nutrient scavenging in those environments. Extracellular nucleases allow many bacteria, including human pathogens like *Serratia marcescens*, *E*. *coli* or *Pseudomonas aeruginosa*, to grow on DNA as sole carbon or nitrogen source [72–75]. Indeed, NucA and NucB enabled *R*. *solanacearum* to use DNA as a sole carbon source. Although *in vitro* growth on DNA was slow, this ability could confer a valuable advantage in the soil where the bacterium must compete for nutrients with other soil inhabitants. In addition, extracellular nucleases could contribute to *R*. *solanacearum* success in other ways. For example, deoxyadenosine and other by-products of NET degradation by Staphylococcal nucleases are toxic to macrophages, which generates a macrophage-free zone around abscesses during *S*. *aureus* infections [69]. It would be interesting to determine if *R*. *solanacearum* nucleases also release toxic byproducts like deoxyadenosine.

In conclusion, we demonstrate for the first time that DNases are virulence factors in a plant pathogen. Their importance derives from the apparently convergent evolution of DNA-based defenses in animal and plants: plant root border cells and animal macrophages both deploy DNA-containing NETs to trap and kill pathogens. In response, animal pathogens and at least one plant pathogen use secreted enzymes to overcome a DNA-based host defense system. We also observed that *R*. *solanacearum* cells trigger release of DNA-containing traps from plant root border cells. An aflagellate *fliC* mutant cannot induce trap release, but the flg22 flagellin peptide alone can do so, suggesting that root border cell traps are a previously unrecognized element of PAMP-triggered plant innate immunity.

## Materials and Methods

### Bacterial strains, plasmids and culture conditions

Strains and plasmids used in this study are listed in S1 Table. *R*. *solanacearum* was grown at 28°C in either Casamino Acids-Peptone-Glucose (CPG) broth or solid TZC (1.8% agar+0.05% tetrazolium chloride) [76]. For DNase activity assays and natural transformation, bacteria were grown in Boucher’s Minimal Medium (BMM) supplemented with 0.2% glucose [77]. *E*. *coli* was grown in Luria-Bertani medium at 37°C. Media were supplemented with antibiotics as needed: kanamycin (25 μg/ml), gentamycin (25 μg/ml) and tetracycline (15 μg/ml).

### Nuclease mutant construction and complementation

Primers used for strain construction are listed in S2 Table. To create deletion mutants of *R*. *solanacearum* strain GMI1000 lacking *nucA* (locus tag Rsc0744) or *nucB* (RSc2452), we designed primers to amplify approximately 500 bp upstream and downstream of each target gene. The resulting fragments were annealed to the pSJG Gm^R^ cassette (for Δ*nucA* construct) or a the pBYJ-Km1 Km^R^ cassette (for Δ*nucB*) using splicing-by-overlap-extension PCR [78]. The resulting deletion constructs were ligated to pCR-Blunt and transformed into *E*. *coli* cells. Plasmids containing correct constructs as verified by PCR were electroporated into *R*. *solanacearum* and putative mutants with the expected antibiotic resistances were confirmed by PCR. A double Δ*nucA/B* mutant was generated by transforming the Δ*nucA* deletion construct in pCR-Blunt into GMI1000 Δ*nucB* and selecting for Km^R^, Gmt^R^ transformants. A type II secretion system-deficient mutant was constructed by natural transformation of GMI1000 with genomic DNA from K100 (K60 *gspM*::Km^R^) [47].

To complement the nuclease mutants we used pRCT-GWY, which integrates into a selectively neutral *attTn7* site in the *R*. *solanacearum* chromosome [79]. Complementation constructs containing the *nucA* or *nucB* ORFs with their putative promoters were ligated into pCR-Blunt, then subcloned into the *Spe*I/*Bgl*II or *Kpn*I/*Avr*II sites of pRCT-GWY to create pRCT-nucAcom and pRCT-nucBcom, respectively. These constructs were naturally transformed into GMI1000 Δ*nucA* and Δ*nucB* [77,80]. Transformants with desired antibiotic resistance profiles were screened for complementation by PCR, nuclease activity assay and qRT-PCR; the confirmed complemented strains were named GMI1000 nucAcom and nucBcom.

For gene expression analyses, the hot phenol-chloroform method [37] was used to extract total RNA from bacterial cultures grown to mid-log phase in CPG and cDNA was synthesized with SuperScript III VILO (Invitrogen, Carlsbad, CA). Expression of *nucA* and *nucB* was measured using quantitative RT-PCR with Power SYBR Green master mix (Invitrogen) in an ABI PRISM 7300 real-time PCR system (Applied Biosystems, Life Technologies, Waltham, MA). Relative transcript abundance was normalized using the reference gene *rplM* [81].

### DNase activity assays

DNase activity was quantified using the DNase Alert Kit (IDT, Coralville, IA). Overnight cultures of *R*. *solanacearum* grown in BMM+0.2% glucose were centrifuged, passed through a 0.2-μm filter, and this cell-free supernatant was incubated with DNase Alert buffer and substrate at 37°C for 3 h according to the manufacturer’s instructions. DNase activity was read as fluorescence signal released by cleavage of a synthetic oligonucleotide with a fluorescent Hex probe at one end and a fluorescence quencher at the other, using a Synergy HT microplate reader (Biotek Instruments, Winooski, VT). Ten units of DNase I (Ambion, Life Technologies, Carlsbad, CA) and uninoculated BMM were used as positive and negative controls, respectively.

### Overexpression and characterization of *R*. *solanacearum* extracellular nucleases

DNA sequences encoding amino acid residues 51–275 of NucA (the 225 amino acids following a predicted trans-membrane domain) and for full-length NucB were amplified from GMI1000 genomic DNA by PCR and cloned into pCR-Blunt, then subcloned into in the pET29b expression vector, in frame with a 6X-His tag sequence (Novagen, EMD Biosciences, Madison, WI). The resulting expression constructs, pET29b-EnucA and pET29b-EnucB, were transformed into the expression strain *E*. *coli* BL21 Star (Invitrogen) and confirmed by colony PCR and sequencing.

Overnight cultures of *E*. *coli* strains carrying the *nucA* and *nucB* overexpression vectors were diluted 1:500 into 1 L of LB+kanamycin, and grown for 3 h at 37°C. Protein expression was induced by adding 1mM IPTG. After an additional 3 h, bacteria were collected by centrifugation and the His-tagged proteins were purified using nickel columns according to the manufacturer’s protocol (Qiagen, Valencia, CA). Purified proteins were analyzed by SDS-PAGE and by Western Blot with anti-His antibody (Invitrogen). Protein concentration was measured using the Bradford assay.

The substrate specificity of purified nucleases was determined by incubating 1 μg of purified nuclease protein in DNase I buffer for 30 min at 37°C with 1 μg of either salmon sperm DNA (Sigma), *R*. *solanacearum* GMI1000 genomic DNA, pea genomic DNA, or supercoiled plasmid pUCGM [82]. Reactions were stopped by adding 0.1 vol 10% SDS and DNA degradation was visualized by electrophoresis in a 1.5% agarose gel.

Cation preference of NucA and NucB was determined using a modified DNase Alert assay, described above. One microgram of purified nuclease was incubated at 37°C for 3 h in PBS with 10 μl DNase Alert substrate and 10 mM of either Mg^2+^, Mn^2+^, Zn^2+^, or DNase Alert buffer. Buffer amended with 10 mM EDTA to sequester cations was used as a negative control.

### Construction of *nucA-phoA* fusion and PhoA assay

We used *phoA* fusions to test for the membrane location of NucA in *E*. *coli*. The *nucA* ORF without its native stop codon was amplified from GMI1000 genomic DNA using PCR primers nucA-phoAF/R and the resulting PCR product was cloned into pCR-Blunt and then subcloned into the *phoA* fusion vector pDSW438 [83] to create pDSW-*nucA-phoA*. The resulting construct was confirmed by PCR and sequencing, then transformed into chemically competent cells of *E*. *coli* KS272 (*phoA-*). PhoA activity was determined by streaking *E*. *coli* strains carrying pDSW438 (empty vector), pDSW439 [83] (positive control) or pDSW-*nucA*-*phoA* onto plates amended with arabinose and X-phos (5-bromo-4-chloro-3-indolyl phosphate) (Sigma-Aldrich, St. Louis, MO). Results were read after overnight incubation at 37°C. Colonies were blue if the PhoA domain was facing the periplasm and white if the PhoA domain was in the cytoplasm.

### Visualization of root border cell extracellular traps

Pea seeds (cv. Little Marvel) were sterilized in full-strength bleach for 30 min, then 95% ethanol for 10 min, followed by five washes with sterile water [84]. Pea seeds were imbibed in water for 6 h, and allowed to germinate on 1% water agar overlaid with sterile filter paper for 2 days in the dark at room temperature.

Root border cells, collected by dipping root tips into sterile water for 5 min, were incubated with 1x10^8^ CFU/ml. *R*. *solanacearum*-GFP for 30 min and then stained with SYTOX Green or DAPI (Life Technologies, Carlsbad, CA) according to the manufacturer’s instructions. Border cells were visualized under an Olympus BX60F5 fluorescence compound microscope (Olympus, Japan).

To determine which *R*. *solanacearum* signals trigger release of plant extracellular traps, we inoculated pea border cells with wild-type bacteria or mutants lacking either EPS (*epsB*), flagellin (*fliC*), or the type III secretion system (*hrpB*) (S1 Table), or with 20 μg/ml of the synthetic *P*. *aeruginosa* flagellin peptide Flg22 (Genscript, Piscataway Township, NJ). The specificity of trap release was tested by inoculating pea root border cells with suspensions of the non-pathogenic bacteria *Sinorhizobium melilotii*, *Pseudomonas aureofaciens*, *P*. *fluorescens* and *E*. *coli*. Approximately 10,000 border cells from 4 pea roots were pooled and used for each biological replicate. Each experiment was repeated three times. After staining with SYTOX Green, the suspensions were observed under a Zeiss Elyra PS.1 LSM 780 confocal laser scanning microscope between 30 and 60 min after inoculation with 10^7^ CFU/ml *R*. *solanacearum*. For each replicate, at least 5 images were taken; representative images are shown. Confocal imaging was performed at the Newcomb Imaging Center, Department of Botany, University of Wisconsin-Madison.

To visualize border cell extracellular traps by scanning electron microscopy (SEM), axenic pea roots were inoculated by dipping the roots into a 10^7^ CFU/ml suspension of *R*. *solanacearum* GMI1000. One hour after inoculation at room temperature, the roots were excised and fixed for 8 h in primary fixative (2.5% glutaraldehyde, 2% formaldehyde, 0.003 M MgCl_2_, 0.003 M CaCl_2_ in 0.05 M PIPES buffer, pH 7). The samples were washed twice with 0.05M PIPES buffer and fixed with secondary fixative (1% OsO_4_ in 0.05M PIPES) overnight, then rinsed twice with 0.05M PIPES, dehydrated in ascending ethanol concentrations ending at 100% and critical point dried. Root samples were sputter coated with gold-palladium (1.8 kV and 6 mA for 60 s) and examined in a Zeiss LEO 1530 high-resolution scanning electron microscope at the Material Sciences Center, University of Wisconsin-Madison.

### Histone killing assay

To measure the bactericidal activity of histone H4, *R*. *solanacearum* GMI1000 cells were grown overnight in CPG, pelleted, washed, and adjusted to an OD_600_ of 1.5 with sterile water. Recombinant human histone H4 (New England Biolabs, Ipswitch, MA) was added to a final concentration of 10, 20, 50 or 100 μg/ml and incubated for 3 h at 28°C. Bacterial viability was then measured using the LIVE/DEAD Baclight kit (Life Technologies) according to the manufacturer’s protocol. The percentage of live cells was determined by plotting the SYTO9/PI fluorescence ratio against a standard curve based on known mixtures of live and dead cells. The experiment was repeated twice, each with three technical replicates.

### Root border cell killing assay

Border cells from four pea roots (approximately 10,000 cells) were inoculated with 10^4^ *R*. *solanacearum* cells, incubated in water at room temperature for 3 h with or without 10 units of DNase I or 10 ng/ml anti-histone H4 antibody, vortexed vigorously, and serially dilution plated on CPG plates. Colonies were counted after 2 days at 28°C to quantify the surviving bacterial population size. The experiment was repeated three times, each with six technical replicates.

### Degradation of root border cell extracellular DNA by purified NucA and NucB

Approximately 5000 pea root border cells collected as described above were placed in each well of a black 96-well plate (Corning Inc., Corning, NY) with or without 5x10^6^ CFU *R*. *solanacearum*. Ten units of DNase I (Ambion) or 10 μg of purified NucA or NucB were added along with 2 nM of SYTOX Green DNA stain and reactions were incubated at 25°C for 6 h and fluorescence was measured with a microplate reader at 485nm excitation/528nm emission. Relative fluorescence units (RFU) in each well at 6 h were normalized to RFU of the same well at the beginning of the assay. The experiment was repeated three times, with four technical replicates in each treatment.

### Root attachment assay

Attachment of *R*. *solanacearum* to pea and tomato roots was measured as described [40], with some modifications. Pea seeds were sterilized as described above. Tomato seeds (cv. Bonny Best) were surface-sterilized by soaking for 10 min in 10% sodium hypochlorite; 5 min in 70% ethanol and washed five times in sterile water. Sterilized seeds were then germinated on 1% water agar plates overlaid with filter paper to avoid dispersal of root border cells from the root tip by free water on the agar surface. After 2 or 3 days, the seedlings were transferred to a water agar plate and root tips were inoculated by gently pipetting 2,500 bacteria/cm root and incubating for 2 h at room temperature. Inoculated roots were excised, washed in sterile water to remove unattached bacteria and blotted dry. Four roots were pooled as a technical replicate, homogenized in sterile water using a PowerLyzer (MoBio), and the bacterial population size attached to the root surface was quantified using serial dilution plating. The experiment was repeated three times with at least five technical replicates per treatment in each experiment. Percent cells attached was calculated as the number of CFU recovered from plates over total bacteria inoculated, and normalized to wild-type attachment.

### Plant virulence assays

Wilt-susceptible tomato plants (cv. Bonny Best) were used to evaluate *R*. *solanacearum* virulence using a naturalistic soil-drenching method that requires the pathogen to locate and infect intact host roots [40]. Briefly, unwounded 21-day old plants were inoculated by pouring onto the soil a suspension of wild-type GMI1000, *ΔnucA*, *ΔnucB* or *ΔnucA/B* to a final concentration of 1x10^7^ CFU/g soil. Plants were maintained in a growth chamber with a 12 h light cycle at 28°C and rated daily over 14 days using a 0–4 disease index scale as follows: 0, no leaf wilted; 1, 1–25% leaves wilted; 2, 26–50% leaves wilted; 3, 51–75% leaves wilted and 4, more than 75% leaves wilted. The experiment was repeated three times and each experiment included 14 plants per treatment.

The ability of bacteria to colonize plants was quantified by sampling the mid-stems of tomato plants soil-drench inoculated as described above with either the wild-type strain or the *ΔnucA/B* mutant. At 2, 4 and 6 days after inoculation, a 100-mg transverse slice was harvested from the mid-stem of each plant, homogenized in 900 μl of sterile water using a PowerLyzer (MoBio, Carlsbad, CA), and bacterial population sizes were quantified using serial dilution plating. The experiment was repeated twice with at least 10 plants per biological replicate.

A root growth assay was used to quantify the effect of *R*. *solanacearum* wild-type strain and exDNase mutants on plant root length. Tomato seeds (cv. Bonny Best) and pea seeds (cv. Little Marvel) were sterilized as described above and germinated in the dark at 28°C or room temperature, respectively. Three-day old tomato seedlings and two-day old pea seedlings were inoculated by dipping root tips for 15 min into bacterial suspensions containing 10^7^ CFU/ml (pea) and 10^6^ CFU/ml (tomato). Inoculated seedlings were placed in germination pouches (Mega International, Newport, MN) and kept at 28°C (tomato) or 25°C (pea). Root systems were imaged 10 days post inoculation. The experiment was repeated twice with 5–6 pea seedlings or 10 tomato seedlings per germination pouch.

### *In planta* competition assay

Twenty-one day old ‘Bonny Best’ tomato plants were inoculated by pouring into each pot 50 ml of a suspension containing a 1:1 ratio of GMI1000-GFP and the *ΔnucA/B* mutant at total density of 10^8^ CFU/ml. When the first wilting symptoms appeared, a 100 mg transverse stem section was excised from each wilted tomato plant, homogenized in 900 μl of sterile water by a PowerLyzer (MoBio, Carlsbad, CA) and serially dilution plated on CPG plates amended with Tet (to select for wild-type), or Kan + Gen (to select for the *ΔnucA/B* strain). The experiment was repeated twice with at least 30 plants per replicate.

### Bacterial growth on DNA as sole carbon source

Overnight CPG cultures of *R*. *solanacearum* were pelleted by centrifugation, washed three times with sterile water, resuspended in BMM to A_600_ = 0.01 and incubated in 96-well microtiter plates with or without 5 μg/ml of salmon sperm DNA (Sigma-Aldrich, St. Louis, MO) as the sole carbon source. Bacterial growth was monitored over 72h as A_600_ using a microplate reader. The experiment was repeated three times with three technical replicates in each experiment.

## Supporting Information

S1 Table Bacterial strains and plasmids used in this study. (DOCX)

S2 Table Primers for mutagenesis, complementation and gene expression analysis. (DOCX)

S1 Video *R*. *solanacearum* wild-type strain GMI1000 was trapped shortly after incubation with pea root border cells. 10^7^ *bacterial* cells were incubated with approximately 10,000 border cells from 2-day old pea seedlings. Trapping was monitored immediately after inoculation at 400X magnification using a Leica DM LB light microscope equipped with a Dino AM-4023XC camera.
(WMV)

S2 Video *R*. *solanacearum* wild-type strain GMI1000 had escaped from trapping 24h after after inoculation with pea root border cells. 10^7^ bacterial cells were incubated with approximately 10,000 border cells from 2-day old pea seedlings. The release of trapped bacteria was monitored 24 h post inoculation at 400X magnification using a Leica DM LB light microscope equipped with a Dino AM-4023XC camera.
(WMV)

S3 Video *R*. *solanacearum* Δ*nucA/B* double nuclease mutant cells remained trapped by pea root border cell NETs up to 24 h post inoculation with pea root border cells. 10^7^ mutant bacterial cells were incubated with approximately 10,000 border cells from 2-day old pea seedlings. Trapping was monitored at 24 h post inoculation at 400X magnification using a Leica DM LB light microscope equipped with a Dino AM-4023XC camera.
(WMV)

S4 Video *R*. *solanacearum* Δ*nucA/B* double nuclease mutant cells were released from trapping by pea root border cell NETs after purified NucA was added. 10^7^ mutant bacterial cells were incubated with approximately 10,000 border cells from 2-day old pea seedlings at room temperature for 24h. Ten μg/ml of purified NucA was added and the cells were incubated at room temperature for an additional hour. The release of trapped bacteria was monitored at 400X magnification, using a Leica DM LB light microscope equipped with a Dino AM-4023XC camera.
(WMV)

S5 Video *R*. *solanacearum* Δ*nucA/B* double nuclease mutant cells were released from trapping by pea root border cell NETs after purified NucB protein was added. 10^7^ mutant bacterial cells were incubated with approximately 10,000 border cells from 2-day old pea seedlings at room temperature for 24 h. Purified NucB was added to a final concentration of 10 μg/ml and the cells were incubated at room temperature for an additional hour. The release of trapped bacteria was monitored at 400X magnification, using by a Leica DM LB light microscope equipped with a Dino AM-4023XC camera.
(WMV)

S1 Fig *R*. *solanacearum* is trapped by tomato border cells. (A) Tomato border cells (arrow head) formed trap in response to *R*. *solanacearum* which can be visualized by Toluidine Blue O staining (white arrows). *R*. *solanacearum* cells can be seen along the trap (black arrows). (B) A close-up view of a tomato border cell trap revealing that traps contain DNA (blue staining with Toluidine Blue O– white arrows) in close association with many *R*. *solanacearum* cells (black arrows). Tomato border cells were collected from axenic seedlings as described in Material and Methods. Pictures were taken approximately 30 min after incubation of tomato border cells with the bacterium.
(TIF)

S2 Fig Induction of pea border cell extracellular trap release by non-pathogenic bacteria. Border cells from pea seedling roots were inoculated with 10^7^ cells of *Pseudomonas aureofaciens* (Pau), *Pseudomonas fluorescens* (Pfl), *Sinorhizobium meliloti* (Sme), *E*.*coli* (Ec) or sterile water and stained with SYTOX Green to visualize DNA (white arrows). Live imaging was performed with a Zeiss Elyra 780 CLSM. At least 5 images per treatment were taken between 30 min-1 h post inoculation. Images are representative of two independent experiments (bar = 50 μm).
(TIF)

S3 Fig *R*. *solanacearum* K60 *fliC* cells did not induce trap release from pea border cells. Approximately 10,000 pea border cells were inoculated with 10^7^ CFU of *R*. *solanacearum* K60 flagellin mutant *fliC*. The cell suspension was stained with SYTOX Green and imaged 45 min post inoculation using a Zeiss Elyra 780 CLSM. Extracellular DNA was not observed even when bacteria were close to border cells (arrow heads: bacterial cells; white arrow: pea root border cell). The experiment was repeated three times with similar results.
(TIF)

S4 Fig Genomic context, mutagenesis and complementation of *R*. *solanacearum* nuclease genes. (A) Map showing the genomic context of two putative extracellular nuclease genes in *R*. *solanacearum* strain GMI1000 and the location of the antibiotic resistance gene cassettes that replaced the *nucA* and *nucB* open reading frames. Arrows indicate open reading frames. (B) and (C) Expression of *nucA* and *nucB* in nuclease mutants and complemented mutant strains (Δ*nucA*+*nucA* and Δ*nucB*+nucB), relative to gene expression levels in wild-type GMI1000. RNA was extracted from bacteria cultured in rich CPG medium to 6x10^8^ CFU/ml. Relative transcript abundance was measured using quantitative RT-PCR and normalized to the reference gene *rplM*. The means of three independent experiments are presented; bars show standard error of the mean. Asterisks indicate differences from wild-type gene expression (one-way ANOVA, ns: *P*>0.05, * *P*≤0.05, *** *P*≤0.001, **** *P*≤0.0001).
(TIF)

S5 Fig Growth of *R*. *solanacearum* in minimal medium with DNA as the sole carbon source. Wild-type *R*. *solanacearum* strain GMI1000 and the *ΔnucA/nucB* double nuclease mutant were grown in minimal medium with or without 5 μg/ml salmon sperm DNA as the sole carbon source. Bacterial growth was measured as absorbance at 600nm using a BioTek plate reader. Strains and growth conditions are indicated as follows: wild-type + DNA, closed circle; *ΔnucA/B* + DNA, closed triangle; wild-type + no DNA, open circle; *ΔnucA/B* + no DNA, open triangle (p<0.005, repeated measures ANOVA).
(TIF)

S6 Fig Overexpression and characterization of NucA and NucB nuclease activity. (A) Overexpression plasmid pET29b containing either the *nucA* or the *nucB* ORF was transformed into *E*. *coli* BL21Star and gene expression was induced with IPTG. The resulting recombinant proteins were purified using nickel columns and detected by Western blot using anti-His antibody (M: 6XHis ladder). (B) Alkaline phosphatase assay of NucA-PhoA fusion in *E*. *coli phoA* ^-^ strain KS272. Blue or white color of the colonies indicates the PhoA domain is facing the periplasm or the cytoplasm, respectively. (C) and (D) DNase activity of purified NucA (C) and NucB (D) in the presence of different cations or DNase Alert buffer + EDTA. Each reaction contained 2 μg of purified NucA or NucB enzyme. DNase activity was measured by DNase Alert assay using a fluorescence plate reader after 3 h incubation at 37°C. Asterisks indicate difference from EDTA treatment (one-way ANOVA, ** *P*<0.01).
(TIF)

S7 Fig A resistant and a susceptible tomato cultivar produced different amounts of root border cells and border cell mucilage. (A) Root border cell counts from 3-day old tomato seedling roots of wilt-susceptible cultivar Bonny Best and wilt-resistant breeding line Hawaii 7996. Seeds from tomato cultivars Hawaii 7996 and Bonny Best were surfaced-sterilized as described above. We germinated the seeds on 1% water agar plates overlaid with filter paper and incubated at 28°C for 4 days. Root border cells were collected by dipping the seedling root tips into sterile water for 1–2 min. The number of border cells was counted under a light microscope as previously described [84]. The experiment was repeated twice, with 10 seedlings for each cultivar. Bars represent manual counts of average border cells from 20 fully germinated seedlings of each cultivar (Student’s t-test, *P*<0.001). (B) To visualize the slime layer produced by border cells, we collected tomato root border cells from the two cultivars and stained the border cell suspension with India Black ink. Images of both single and clusters of border cells were taken with a light microscope.
(TIF)

## References

[1] Wen F, VanEtten HD, Tsaprailis G, Hawes MC (2007) Extracellular proteins in pea root tip and border cell exudates. Plant Physiology 143: 773–783. 17142479 10.1104/pp.106.091637 PMC1803736

[2] Wen F, White GJ, VanEtten HD, Xiong Z, Hawes MC (2009) Extracellular DNA is required for root tip resistance to fungal infection. Plant Physiology 151: 820–829. 10.1104/pp.109.142067 19700564 PMC2754639

[3] Pietramellara G, Ascher J, Borgogni F, Ceccherini M, Guerri G, et al (2009) Extracellular DNA in soil and sediment: fate and ecological relevance. Biology and Fertility of Soils 45: 219–235.

[4] Paget E, Lebrun M, Freyssinet G, Simonet P (1998) The fate of recombinant plant DNA in soil. European Journal of Soil Biology 34: 81–88.

[5] Wackernagel W (2006) The various sources and the fate of nucleic acids in soil In: Nannipieri P, Smalla K, editors. Nucleic acids and proteins in soil. Verlag-Berlin-Heidelberg: Springer pp. 117–139.

[6] de Vries J, Heine M, Harms K, Wackernagel W (2003) Spread of recombinant DNA by roots and pollen of transgenic potato plants, identified by highly specific biomonitoring using natural transformation of an *Acinetobacter* sp. Applied and environmental microbiology 69: 4455–4462. 12902229 10.1128/AEM.69.8.4455-4462.2003 PMC169075

[7] Hawes MC, Curlango-Rivera G, Wen F, White GJ, Vanetten HD, et al (2011) Extracellular DNA: the tip of root defenses? Plant Sci 180: 741–745. 10.1016/j.plantsci.2011.02.007 21497709

[8] Brinkmann V, Reichard U, Goosmann C, Fauler B, Uhlemann Y, et al (2004) Neutrophil extracellular traps kill bacteria. Science 303: 1532–1535. 15001782 10.1126/science.1092385

[9] Urban CF, Reichard U, Brinkmann V, Zychlinsky A (2006) Neutrophil extracellular traps capture and kill *Candida albicans* yeast and hyphal forms. Cell Microbiol 8: 668–676. 16548892 10.1111/j.1462-5822.2005.00659.x

[10] Urban CF, Lourido S, Zychlinsky A (2006) How do microbes evade neutrophil killing? Cell Microbiol 8: 1687–1696. 16939535 10.1111/j.1462-5822.2006.00792.x

[11] Halverson TWR, Wilton M, Poon KKH, Petri B, Lewenza S (2015) DNA is an antimicrobial component of neutrophil extracellular traps. PLoS Pathog 11: e1004593 10.1371/journal.ppat.1004593 25590621 PMC4295883

[12] Yipp BG, Petri B, Salina D, Jenne CN, Scott BNV, et al (2012) Infection-induced NETosis is a dynamic process involving neutrophil multitasking *in vivo* . Nature Medicine 18: 1386–1393. 22922410 10.1038/nm.2847 PMC4529131

[13] Remijsen Q, Kuijpers T, Wirawan E, Lippens S, Vandenabeele P, et al (2011) Dying for a cause: NETosis, mechanisms behind an antimicrobial cell death modality. Cell Death and Differentiation 18: 581–588. 10.1038/cdd.2011.1 21293492 PMC3131909

[14] Wartha F, Beiter K, Albiger B, Fernebro J, Zychlinsky A, et al (2007) Capsule and d-alanylated lipoteichoic acids protect *Streptococcus pneumoniae* against neutrophil extracellular traps. Cell Microbiol 9: 1162–1171. 17217430 10.1111/j.1462-5822.2006.00857.x

[15] Beiter K, Wartha F, Albiger B, Normark S, Zychlinsky A, et al (2006) An endonuclease allows *Streptococcus pneumoniae* to escape from neutrophil extracellular traps. Curr Biol 16: 401–407. 16488875 10.1016/j.cub.2006.01.056

[16] Derré-Bobillot A, Cortes-Perez NG, Yamamoto Y, Kharrat P, Couvé E, et al (2013) Nuclease A (Gbs0661), an extracellular nuclease of *Streptococcus agalactiae*, attacks the neutrophil extracellular traps and is needed for full virulence. Molecular Microbiology 89: 518–531. 10.1111/mmi.12295 23772975

[17] Sumby P, Barbian KD, Gardner DJ, Whitney AR, Welty DM, et al (2005) Extracellular deoxyribonuclease made by group A *Streptococcus* assists pathogenesis by enhancing evasion of the innate immune response. Proceedings of the National Academy of Sciences, USA 102: 1679–1684. 10.1073/pnas.0406641102 PMC547841 15668390

[18] Buchanan JT, Simpson AJ, Aziz RK, Liu GY, Kristian SA, et al (2006) DNase expression allows the pathogen group A *Streptococcus* to escape killing in neutrophil extracellular traps. Curr Biol 16: 396–400. 16488874 10.1016/j.cub.2005.12.039

[19] Thammavongsa V, Missiakas DM, Schneewind O (2013) *Staphylococcus aureus* degrades neutrophil extracellular traps to promote immune cell death. Science 342: 863–866. 10.1126/science.1242255 24233725 PMC4026193

[20] Seper A, Hosseinzadeh A, Gorkiewicz G, Lichtenegger S, Roier S, et al (2013) *Vibrio cholerae* evades neutrophil extracellular traps by the activity of two extracellular nucleases. PLoS Pathog 9: e1003614 10.1371/journal.ppat.1003614 24039581 PMC3764145

[21] Juneau RA, Stevens JS, Apicella MA, Criss AK (2015) A thermonuclease of *Neisseria gonorrhoeae* enhances bacterial escape from killing by neutrophil extracellular traps. Journal of Infectious Diseases 212: 316–324. 10.1093/infdis/jiv031 25605868 PMC4490236

[22] Guimarães-Costa AB, DeSouza-Vieira TS, Paletta-Silva R, Freitas-Mesquita AL, Meyer-Fernandes JR, et al (2014) 3′-nucleotidase/nuclease activity allows *Leishmania* parasites to escape killing by neutrophil extracellular traps. Infection and immunity 82: 1732–1740. 10.1128/IAI.01232-13 24516114 PMC3993383

[23] Driouich A, Follet-Gueye M-L, Vicré-Gibouin M, Hawes M (2013) Root border cells and secretions as critical elements in plant host defense. Current Opinion in Plant Biology 16: 489–495. 10.1016/j.pbi.2013.06.010 23856080

[24] Gerhold DL, Pettinger AJ, Hadwiger LA (1993) Characterization of a plant-simulated nuclease from *Fusarium solani* . Physiological and Molecular Plant Pathology 43: 33–46.

[25] Hadwiger LA, Druffel K, Humann JL, Schroeder BK (2013) Nuclease released by *Verticillium dahliae* is a signal for non-host resistance. Plant Science 201–202: 98–107. 10.1016/j.plantsci.2012.11.011 23352407

[26] Denny T (2006) Plant pathogenic *Ralstonia* species In: Gnanamanickam SS, editor. Plant-associated bacteria. Dordrecht, The Netherlands: Springer pp. 573–644.

[27] Hayward AC (1991) Biology and epidemiology of bacterial wilt caused by *Pseudomonas solanacearum* . Annu Rev Phytopathol 29: 65–87. 18479193 10.1146/annurev.py.29.090191.000433

[28] Yao J, Allen C (2006) Chemotaxis is required for virulence and competitive fitness of the Bacterial Wilt pathogen *Ralstonia solanacearum* . J Bacteriol 188: 3697–3708. 16672623 10.1128/JB.188.10.3697-3708.2006 PMC1482862

[29] Tans-Kersten J, Huang H, Allen C (2001) *Ralstonia solanacearum* needs motility for invasive virulence on tomato. J Bacteriol 183: 3597–3605. 11371523 10.1128/JB.183.12.3597-3605.2001 PMC95236

[30] McGarvey J, Denny T, Schell M (1999) Spatial-temporal and quantitative analysis of growth and EPS I production by *Ralstonia solanacearum* in resistant and susceptible tomato cultivars. Phytopathology 89: 1233–1239. 10.1094/PHYTO.1999.89.12.1233 18944650

[31] Saile E, McGarvey JA, Schell MA, Denny TP (1997) Role of extracellular polysaccharide and endoglucanase in root invasion and colonization of tomato plants by *Ralstonia solanacearum* . Phytopathology 87: 1264–1271. 10.1094/PHYTO.1997.87.12.1264 18945028

[32] Genin S, Denny TP (2012) Pathogenomics of the *Ralstonia solanacearum* species complex. Annu Rev Phytopathol 50: 67–89. 10.1146/annurev-phyto-081211-173000 22559068

[33] Milling A, Babujee L, Allen C (2011) *Ralstonia solanacearum* extracellular polysaccharide is a specific elicitor of defense responses in wilt-resistant tomato plants. PLoS ONE 6: e15853 10.1371/journal.pone.0015853 21253019 PMC3017055

[34] Genin S, Gough CL, Zischek C, Boucher CA (1992) Evidence that the *hrpB* gene encodes a positive regulator of pathogenicity genes from *Pseudomonas solanacearum* . Molecular microbiology 6: 3065–3076. 1479894 10.1111/j.1365-2958.1992.tb01764.x

[35] Brinkmann V, Zychlinsky A (2012) Neutrophil extracellular traps: Is immunity the second function of chromatin? Journal of Cell Biology 198: 773–783. 10.1083/jcb.201203170 22945932 PMC3432757

[36] Wen F, Curlango-Rivera G, Hawes MC (2007) Proteins among the polysaccharides: a new perspective on root cap 'slime'. Plant Signaling and Behavior 2: 410–412. 19704617 10.4161/psb.2.5.4344 PMC2634230

[37] Jacobs JM, Babujee L, Meng F, Milling A, Allen C (2012) The *in planta* transcriptome of *Ralstonia solanacearu*m: conserved physiological and virulence strategies during bacterial wilt of tomato. MBio 3: e00114–00112. 10.1128/mBio.00114-12 22807564 PMC3413399

[38] Guidot A, Prior P, Schoenfeld J, Carrere S, Genin S, et al (2007) Genomic structure and phylogeny of the plant pathogen *Ralstonia solanacearum* inferred from gene distribution analysis. J Bacteriol 189: 377–387. 17085551 10.1128/JB.00999-06 PMC1797399

[39] Liu H, Zhang S, Schell MA, Denny TP (2005) Pyramiding unmarked deletions in *Ralstonia solanacearum* shows that secreted proteins in addition to plant cell-wall-degrading enzymes contribute to virulence. Molecular plant-microbe interactions 18: 1296–1305. 16478049 10.1094/MPMI-18-1296

[40] Yao J, Allen C (2007) The plant pathogen *Ralstonia solanacearum* needs aerotaxis for normal biofilm formation and interactions with its tomato host. Journal of Bacteriology 189: 6415–6424. 17601784 10.1128/JB.00398-07 PMC1951909

[41] Curlango-Rivera G, Huskey D, Mostafa A, Kessler JO, Xiong Z, et al (2012) Increased border cell numbers in cotton: rhizosphere microbiome implications. American Journal of Botany 100: 1706–1712. 10.3732/ajb.1200607 23942085

[42] Gochnauer M, Sealey L, McCully M (1990) Do detached root-cap cells influence bacteria associated with maize roots? Plant Cell and Environment 13: 793–801.

[43] Gunawardena U, Hawes MC (2002) Tissue specific localization of root infection by fungal pathogens: role of root border cells. Molecular plant-microbe interactions 15: 1128–1136. 12423018 10.1094/MPMI.2002.15.11.1128

[44] Plancot B, Santaella C, Jaber R, Kiefer-Meyer MC, Follet-Gueye M-L, et al (2013) Deciphering the responses of root border-like cells of *Arabidopsis* and flax to pathogen-derived elicitors. Plant physiology 163: 1584–1597. 10.1104/pp.113.222356 24130195 PMC3850203

[45] Rosli HG, Zheng Y, Pombo MA, Zhong S, Bombarely A, et al (2013) Transcriptomics-based screen for genes induced by flagellin and repressed by pathogen effectors identifies a cell wall-associated kinase involved in plant immunity. Genome biology 14: 1–15. 10.1186/gb-2013-14-12-r139 PMC4053735 24359686

[46] Jones JD, Dangl JL (2006) The plant immune system. Nature 444: 323–329. 17108957 10.1038/nature05286

[47] Pfund C, Tans-Kersten J, Dunning FM, Alonso JM, Ecker JR, et al (2004) Flagellin is not a major defense elicitor in *Ralstonia solanacearum* cells or extracts applied to *Arabidopsis thaliana* . Molecular Plant-microbe interactions 17: 696–706. 15195952 10.1094/MPMI.2004.17.6.696

[48] Clarke CR, Chinchilla D, Hind SR, Taguchi F, Miki R, et al (2013) Allelic variation in two distinct *Pseudomonas syringae* flagellin epitopes modulates the strength of plant immune responses but not bacterial motility. New Phytologist 200: 847–860. 10.1111/nph.12408 23865782 PMC3797164

[49] Wood TK, Barrios AFG, Herzberg M, Lee J (2006) Motility influences biofilm architecture in *Escherichia coli* . Applied Microbiology and Biotechnology 72: 361–367. 16397770 10.1007/s00253-005-0263-8

[50] Brinkmann V, Goosmann C, Kühn LI, Zychlinsky A (2012) Automatic quantification of *in vitro* NET formation. Frontiers in Immunology 3: 1–8. 23316198 10.3389/fimmu.2012.00413 PMC3540390

[51] Wang Y, Li M, Stadler S, Correll S, Li P, et al (2009) Histone hypercitrullination mediates chromatin decondensation and neutrophil extracellular trap formation. Journal of Cell Biology 184: 205–213. 10.1083/jcb.200806072 19153223 PMC2654299

[52] Patel S, Kumar S, Jyoti A, Srinag BS, Keshari RS, et al (2010) Nitric oxide donors release extracellular traps from human neutrophils by augmenting free radical generation. Nitric Oxide 22: 226–234. 10.1016/j.niox.2010.01.001 20060922

[53] Wendehenne D, Durner J, Klessig D (2004) Nitric oxide: a new player in plant signalling and defence responses. Current opinion in plant biology 7: 449–455. 15231269 10.1016/j.pbi.2004.04.002

[54] Brinkmann V, Zychlinsky A (2007) Beneficial suicide: why neutrophils die to make NETs. Nature Reviews: Microbiology 5: 577–582. 17632569 10.1038/nrmicro1710

[55] Papayannopoulos V, Metzler KD, Hakkim A, Zychlinsky A (2010) Neutrophil elastase and myeloperoxidase regulate the formation of neutrophil extracellular traps. Journal of Cell Biology 191: 677–691. 10.1083/jcb.201006052 20974816 PMC3003309

[56] Remijsen Q, Berghe TV, Wirawan E, Asselbergh B, Parthoens E, et al (2011) Neutrophil extracellular trap cell death requires both autophagy and superoxide generation. Cell Research 21: 290–304. 10.1038/cr.2010.150 21060338 PMC3193439

[57] Chandok MR, Sopory SK (1996) Identification of phorbol myristate acetate stimulated kinase in *Zea mays* . Journal of Plant Biochemistry and Biotechnology 5: 7–11.

[58] Pitzschke A, Schikora A, Hirt H (2009) MAPK cascade signalling networks in plant defence. Current opinion in plant biology 12: 421–426. 10.1016/j.pbi.2009.06.008 19608449

[59] Hirsch JG (1958) Bactericidal action of histone. Journal of Experimental Medicine 108: 925–944. 13598820 10.1084/jem.108.6.925 PMC2136925

[60] Watson BS, Bedair MF, Urbanczyk-Wochniak E, Huhman DV, Yang DS, et al (2015) Integrated metabolomics and transcriptomics reveal enhanced specialized metabolism in *Medicago truncatula* root border cells. Plant physiology 167: 1699–1716. 10.1104/pp.114.253054 25667316 PMC4378151

[61] Hawes M, Brigham L, Wen F, Woo H, Zhu Y (1998) Function of root border cells in plant health: Pioneers 1 in the rhizosphere. Annual Review of Phytopathology 36: 311–327. 15012503 10.1146/annurev.phyto.36.1.311

[62] Hamamoto L, Hawes MC, Rost TL (2006) The production and release of living root cap border cells is a function of root apical meristem type in dicotyledonous angiosperm plants. Ann Bot 97: 917–923. 16488922 10.1093/aob/mcj602 PMC2803423

[63] Gerhold DL, Pettinger AJ, Hadwiger LA (1993) Characterization of a plant-stimulated nuclease from *Fusarium solani* . Physiol Mol Plant Pathol 43: 33–46.

[64] Ma L-S, Hachani A, Lin J-S, Filloux A, Lai E-M (2014) *Agrobacterium tumefaciens* deploys a superfamily of type VI secretion DNase effectors as weapons for interbacterial competition *in planta* . Cell Host and Microbe 16: 94–104. 10.1016/j.chom.2014.06.002 24981331 PMC4096383

[65] Moulard M, Condemine G, Robert-Baudouy J (1993) Search for the function of the nuclease NucM of *Erwinia chrysanthemi* . FEMS Microbiology Letters 112: 99–103.

[66] Álvarez B, Vasse J, Le-Courtois V, Trigalet-Demery D, López MM, et al (2008) Comparative behavior of *Ralstonia solanacearum* biovar 2 in diverse plant species. Phytopathology 98: 59–68. 10.1094/PHYTO-98-1-0059 18943239

[67] Lazarides E, Lindberg U (1974) Actin is the naturally occurring inhibitor of deoxyribonuclease I. Proceedings of the National Academy of Sciences of the United States of America 71: 4742–4746. 4140510 10.1073/pnas.71.12.4742 PMC433972

[68] Macho AP, Guidot A, Barberis P, Beuzón CR, Genin S (2010) A competitive index assay identifies several *Ralstonia solanacearum* type III effector mutant strains with reduced fitness in host plants. Molecular Plant-Microbe Interactions 23: 1197–1205. 10.1094/MPMI-23-9-1197 20687809

[69] Papayannopoulos V (2014) Infection: microbial nucleases turn immune cells against each other. Current Biology 24: R123–R125. 10.1016/j.cub.2013.12.027 24502788

[70] Dell'Anno A, Corinaldesi C (2004) Degradation and turnover of extracellular DNA in marine sediments: ecological and methodological considerations. Applied and environmental microbiology 70: 4384–4386. 15240325 10.1128/AEM.70.7.4384-4386.2004 PMC444808

[71] Lorenz M, Aardema B, Krumbein W (1981) Interaction of marine sediment with DNA and DNA availability to nucleases. Marine Biology 64: 225–230.

[72] Beliaeva M, Kapranova M, Vitol M, Golubenko I, Leshchinskaia I (1975) Nucleic acids utilized as the main source of bacterial nutrition. Mikrobiologiia 45: 420–424. 826761

[73] Pinchuk GE, Ammons C, Culley DE, Li SM, McLean JS, et al (2008) Utilization of DNA as a sole source of phosphorus, carbon, and energy by *Shewanella* spp.: ecological and physiological implications for dissimilatory metal reduction. Appl Environ Microbiol 74: 1198–1208. 18156329 10.1128/AEM.02026-07 PMC2258558

[74] Palchevskiy V, Finkel S (2009) A role for single-stranded exonucleases in the use of DNA as a nutrient. Journal of bacteriology 191: 3712–3716. 10.1128/JB.01678-08 19329645 PMC2681894

[75] Mulcahy H, Charron-Mazenod L, Lewenza S (2010) *Pseudomonas aeruginosa* produces an extracellular deoxyribonuclease that is required for utilization of DNA as a nutrient source. Environmental Microbiology 12: 1621–1629. 10.1111/j.1462-2920.2010.02208.x 20370819

[76] Kelman A (1954) The relationship of pathogenicity of *Pseudomonas solanacearum* to colony appearance in a tetrazolium medium. Phytopathology 44: 693–695.

[77] Boucher CA, Barberis PA, Demery DA (1985) Transposon mutagenesis of *Pseudomonas solanacearu*m: isolation of Tn5-induced avirulent mutants. Journal of General Microbiology 131: 2449–2457.

[78] Horton R, Hunt H, Ho S, Pullen J, Pease L (1989) Engineering hybrid genes without the use of restriction enzymes: gene splicing by overlap extension. Gene 77: 61–68. 2744488 10.1016/0378-1119(89)90359-4

[79] Monteiro F, Sole M, van Dijk I, Valls M (2012) A chromosomal insertion toolbox for promoter probing, mutant complementation, and pathogenicity studies in *Ralstonia solanacearum* . Mol Plant Microbe Interact 25: 557–568. 10.1094/MPMI-07-11-0201 22122329

[80] Bertolla F, Van Gijsegem F, Nesme X, Simonet P (1997) Conditions for natural transformation of *Ralstonia solanacearum* . Applied and environmental microbiology 63: 4965–4968. 9406418 10.1128/aem.63.12.4965-4968.1997 PMC168825

[81] Monteiro F, Genin S, van Dijk I, Valls M (2012) A luminescent reporter evidences active expression of *Ralstonia solanacearum* type III secretion system genes throughout plant infection. Microbiology 158: 2107–2116. 10.1099/mic.0.058610-0 22609750

[82] Gerceker D, Karasartova D, Elyürek E, Barkar S, KIyan M, et al (2009) A new, simple, rapid test for detection of DNase activity of microorganisms: DNase Tube test. Journal of General and Applied Microbiology 55: 291–294. 19700923 10.2323/jgam.55.291

[83] Arends SR, Kustusch RJ, Weiss DS (2009) ATP-binding site lesions in FtsE impair cell division. Journal of bacteriology 191: 3772–3784. 10.1128/JB.00179-09 19376877 PMC2698383

[84] Hawes MC, Lin H-J (1990) Correlation of pectolytic enzyme activity with the programmed release of cells from root caps of pea (*Pisum sativum*). Plant Physiology 94: 1855–1859. 16667927 10.1104/pp.94.4.1855 PMC1077464

