# Supplementary material for: Escaping Underground Nets: Extracellular DNases Degrade Plant Extracellular Traps and Contribute to Virulence of the Plant Pathogenic Bacterium Ralstonia solanacearum
Source: PLoS Pathog. 2016 Jun 23;12(6):e1005686. doi: 10.1371/journal.ppat.1005686 (PMC4919084; doi:10.1371/journal.ppat.1005686)
Supplement: S1 Table — (DOCX) [file ppat.1005686.s001.docx]

**S1 Table.** Bacterial strains and plasmids used in this study

| **Strains** | **Relevant characteristics** | **Source** | |
| --- | --- | --- | --- |
| ***E. coli*** | | |  |
| TOP10 | F- *mcrA* Δ(*mrr-hsdRMS-mcrBC*) *φ80lacZ*ΔM15 *Δlac*X*74 nupG recA1 araD139* Δ(*ara-leu*)*7697 galE15 galK16 rpsL*(Str^R^) *endA1 λ^-^* | Invitrogen | |
| DH5α | F– Φ80*lac*ZΔM15 Δ(*lac*ZYA-argF) U169 *rec*A1 *end*A1 *hsd*R17 (rK–, mK+) *pho*A *sup*E44 λ– *thi*-1 *gyr*A96 *rel*A1 | Invitrogen | |
| BL21Star | F- *ompT hsdSB (rB-mB-) gal dcm rne131* (DE3) | Invitrogen | |
| KS272 | *phoA*^-^ host strain | [1] | |
| *ftsI-phoA* | KS272 with the *ftsI-phoA* construct in pDSW438 | [2] | |
| ***R. solanacearum*** |  |  | |
| GMI1000 | Wild-type tomato isolate, phylotype I |  | |
| GMI1000 Δ*nucA* | Δ*nucA*::*aacC1*, Gen^R^ | This study | |
| GMI1000 Δ*nucB* | Δ*nucB*::*nptII*, Kan^R^ | This study | |
| GMI1000 Δ*nucA/B* | Δ*nucA*::*aacC*, Δ*nucB*::*nptII*, Gen^R^, Kan^R^ | This study | |
| GMI1000 Δ*nucA*+*nucA* | GMI1000 Δ*nucA* transformed with pRCT-nucAcom | This study | |
| GMI1000 Δ*nucB*+*nucB* | GMI1000 Δ*nucB* transformed with pRCT-nucBcom | This study | |
| K60 *gfp* | GFP-tagged K60, Tet^R^ | [3] | |
| GMI1000 *gfp* | Wild-type GMI1000 transformed with K60 *gfp* DNA, Tet^R^ | This study | |
| K100 | K60 *gspM*::Km, Kan^R^ | [4] | |
| GMI1000 *gspM* | GMI1000 transformed with K100 genomic DNA, Kan^R^ | This study | |
| GMI1000 *hrpB* | *hrpB* mutant, Gen^R^ | [5] | |
| K701 | *fliC* mutant (K701), Gen^R^ | [6] | |
| UW551 *epsB* | *epsB* mutant, Kan^R^ | [7] | |
| **Non-pathogenic bacteria** | | |  |
| *Pseudomonas fluorescens* |  | Allen lab collection | |
| *Pseudomonas aureofaciens* |  | Allen lab collection | |
| *Sinorhizobium meliloti* 2011 |  | [8] | |
| **Plasmids** |  |  | |
| pCR-Blunt | Blunt-end cloning vector, Kan^R^ | Invitrogen | |
| pET29b | *E. coli* expression vector | Novagen | |
| pRCT-GWY | Complementation vector, Tet^R^ | [9] | |
| pSJG | Gentamycin resistance cassette, Gen^R^ | [3] | |
| pBYJ-Km1 | Kanamycin resistance cassette, Kan^R^ | [3] | |
| pDSW438 | pBAD33::’*phoA* | [2] | |
| pDSW-*nucA* | Full-length *nucA* cloned into pDSW438 | This study | |
| pET29b-EnucA | Truncated *nucA* cloned into pET29b | This study | |
| pET29b-EnucB | Full-length *nucB* cloned into pET29b | This study | |
| pCR-*nucA::aacC1* | Δ*nucA*::*aacC1* construct in pCR-Blunt, Gen^R^, Kan^R^ | This study | |
| pCR-*nucB::nptII* | Δ*nucB*::*nptII* construct in pCR-Blunt, Kan^R^ | This study | |
| pRCT-nucAcom | pRCT-GWY containing *nuc*A complementation construct, Tet^R^ | This study | |
| pRCT-nucBcom | pRCT-GWY containing *nuc*B complementation construct, Tet^R^ | This study | |

**S1 Table References**

1. Strauch KL, Beckwith J (1988) An *Escherichia coli* mutation preventing degradation of abnormal periplasmic proteins. Proceedings of the National Academy of Sciences of the United States of America 85: 1576-1580.

2. Arends SR, Kustusch RJ, Weiss DS (2009) ATP-binding site lesions in FtsE impair cell division. Journal of bacteriology 191: 3772-3784.

3. Yao J, Allen C (2006) Chemotaxis is required for virulence and competitive fitness of the Bacterial Wilt pathogen *Ralstonia solanacearum*. J Bacteriol 188: 3697-3708.

4. Pfund C, Tans-Kersten J, Dunning FM, Alonso JM, Ecker JR, et al. (2004) Flagellin is not a major defense elicitor in *Ralstonia solanacearum* cells or extracts applied to *Arabidopsis thaliana*. Molecular Plant-microbe interactions 17: 696-706.

5. Genin S, Gough CL, Zischek C, Boucher CA (1992) Evidence that the *hrpB* gene encodes a positive regulator of pathogenicity genes from *Pseudomonas solanacearum*. Molecular microbiology 6: 3065-3076.

6. Tans-Kersten J, Huang H, Allen C (2001) *Ralstonia solanacearum* needs motility for invasive virulence on tomato. J Bacteriol 183: 3597-3605.

7. Milling A, Babujee L, Allen C (2011) *Ralstonia solanacearum* extracellular polysaccharide is a specific elicitor of defense responses in wilt-resistant tomato plants. PLoS ONE 6: e15853.

8. Sallet E, Roux B, Sauviac L, Carrère S, Faraut T, et al. (2013) Next-generation annotation of prokaryotic genomes with EuGene-P: application to *Sinorhizobium meliloti* 2011. DNA Research 20: 339-354.

9. Monteiro F, Sole M, van Dijk I, Valls M (2012) A chromosomal insertion toolbox for promoter probing, mutant complementation, and pathogenicity studies in *Ralstonia solanacearum*. Mol Plant Microbe Interact 25: 557-568.
