# Supplementary material for: Escaping Underground Nets: Extracellular DNases Degrade Plant Extracellular Traps and Contribute to Virulence of the Plant Pathogenic Bacterium Ralstonia solanacearum
Source: PLoS Pathog. 2016 Jun 23;12(6):e1005686. doi: 10.1371/journal.ppat.1005686 (PMC4919084; doi:10.1371/journal.ppat.1005686)
Supplement: S2 Table — (DOCX) [file ppat.1005686.s002.docx]

**Table.** Primers for mutagenesis, complementation and gene expression analysis

| **Primers** | **Sequence (5’-3’)** | **Source** |
| --- | --- | --- |
| *Mutagenesis* | | |
| nucAUF | GCGGCAAGAAGGGACCGAAC | This study |
| nucAUR | CAATTCGAGCTCGGTACCCCGATGCCAATCGGCGCACTTC | This study |
| nucADF | AAATTGTCACAACGCCGCGGGCGTTATCCTGTGCGGCCATGT | This study |
| nucADR | TGCAGGCAAGCATCAGGTTGCG | This study |
| nucADR | TGCAGGCAAGCATCAGGTTGCG | This study |
| nucBUF | GACCAGCACCAGCATCCCGTG | This study |
| nucBUR | CCCAATTCGCCCTATAGTGAGCTCCGATGAATGGATTGCCGT | This study |
| nucBDF | CGCTCACAATTCCACACAACAAGCCGTCGGTCTGGTGGACA | This study |
| nucBDR | GTCCAACGTGCATCCGGCCC | This study |
| GmF | GGGTACCGAGCTCGAATTG | [1] |
| GmR | CCGCGGCGTTGTGACAATTT | [1] |
| KmF | TCACTATAGGGCGAATTGGG | [2] |
| KmR | GTTGTGTGGAATTGTGAGCG | [2] |
| M13F | GTAAAACGACGGCCAG | Invitrogen |
| M13R | CAGGAACAGCTATGAC | Invitrogen |
| *Complementation* |  |  |
| nucAcF | NN**ACTAGT**GGGGATCACCATGCTGGTTT | This study |
| nucAcR | NN**AGATCT**CAACGAAGGAAAGCGCAGAT | This study |
| nucBcF | NN**CCTAGG**AGACTATGGGGGAAAACCGC | This study |
| nucBcR | NN**GGTACC**GCAACGGTCCATTTCGTGAC | This study |
| *Gene expression analysis* | | |
| nucAqF | GGTAGGAAATCGGTCGGCTC | This study |
| nucAqR | GTATGGGTACCGCACTACCTG | This study |
| nucBqF | CGGTTAGTCAGGCAGGTACG | This study |
| nucBqR | CGCACAACAAGGTCATCGTG | This study |
| qrplM-F | CCGCAAAGCCCCATGAG | [3] |
| qrplM-R | TGTCCGTCGCGTCAATCA | [3] |
| *Protein overexpression* | | |
| EnucAF | NNN**CATATG**CGACCACCGGCTTTGAGCGG | This study |
| EnucAR | CT**CTCGAG**GTTGGCGACCGGCACCCCGG | This study |
| EnucBF | CT**CATATG**ATGAGGACGCGCGGGCTCGG | This study |
| EnucBR | CT**CTCGAG**GTCAGGCAGGTACGGCAGCG | This study |
| T7-promoter | TAATACGACTCACTATAGGG | Novagen |
| T7-terminator | GCTAGTTATTGCTCAGCGG | Novagen |
| *PhoA assay* |  |  |
| nucA-phoAF | NNN**GAGCTC**AAAGGAACCCATGCGCCTGATCCCGCGCGG | This study |
| nucA-phoAR | NNNT**CTAGAG**TTGGCGACCGGCACCCCGG | This study |

**S2 Table References**

1. Dalsing BL, Allen C (2014) Nitrate assimilation contributes to *Ralstonia solanacearum* root attachment, stem colonization, and virulence. Journal of bacteriology 196: 949-960.

2. Jacobs JM, Babujee L, Meng F, Milling A, Allen C (2012) The *in planta* transcriptome of *Ralstonia solanacearu*m: conserved physiological and virulence strategies during bacterial wilt of tomato. MBio 3: e00114-00112.

3. Monteiro F, Sole M, van Dijk I, Valls M (2012) A chromosomal insertion toolbox for promoter probing, mutant complementation, and pathogenicity studies in *Ralstonia solanacearum*. Mol Plant Microbe Interact 25: 557-568.
