# Supplementary figures and images for: Escaping Underground Nets: Extracellular DNases Degrade Plant Extracellular Traps and Contribute to Virulence of the Plant Pathogenic Bacterium Ralstonia solanacearum
Source: PLoS Pathog. 2016 Jun 23;12(6):e1005686. doi: 10.1371/journal.ppat.1005686 (PMC4919084; doi:10.1371/journal.ppat.1005686)

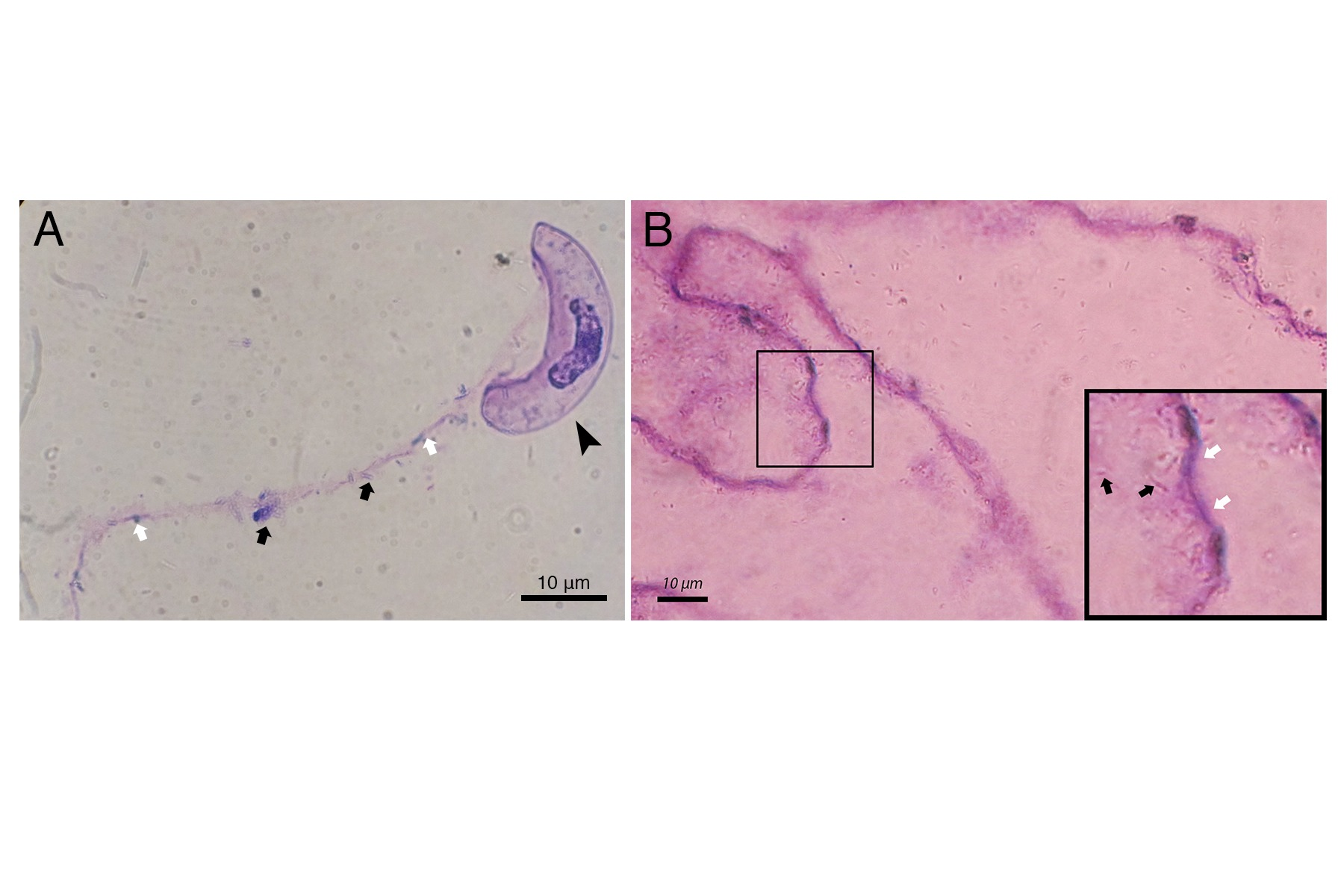

Supplement: S1 Fig — (A) Tomato border cells (arrow head) formed trap in response to R. solanacearum which can be visualized by Toluidine Blue O staining (white arrows). R. solanacearum cells can be seen along the trap (black arrows). (B) A close-up view of a tomato border cell trap revealing that traps contain DNA (blue staining with Toluidine Blue O– white arrows) in close association with many R. solanacearum cells (black arrows). Tomato border cells were collected from axenic seedlings as described in Material and Methods. Pictures were taken approximately 30 min after incubation of tomato border cells with the bacterium. (TIF) [file ppat.1005686.s008.tif]

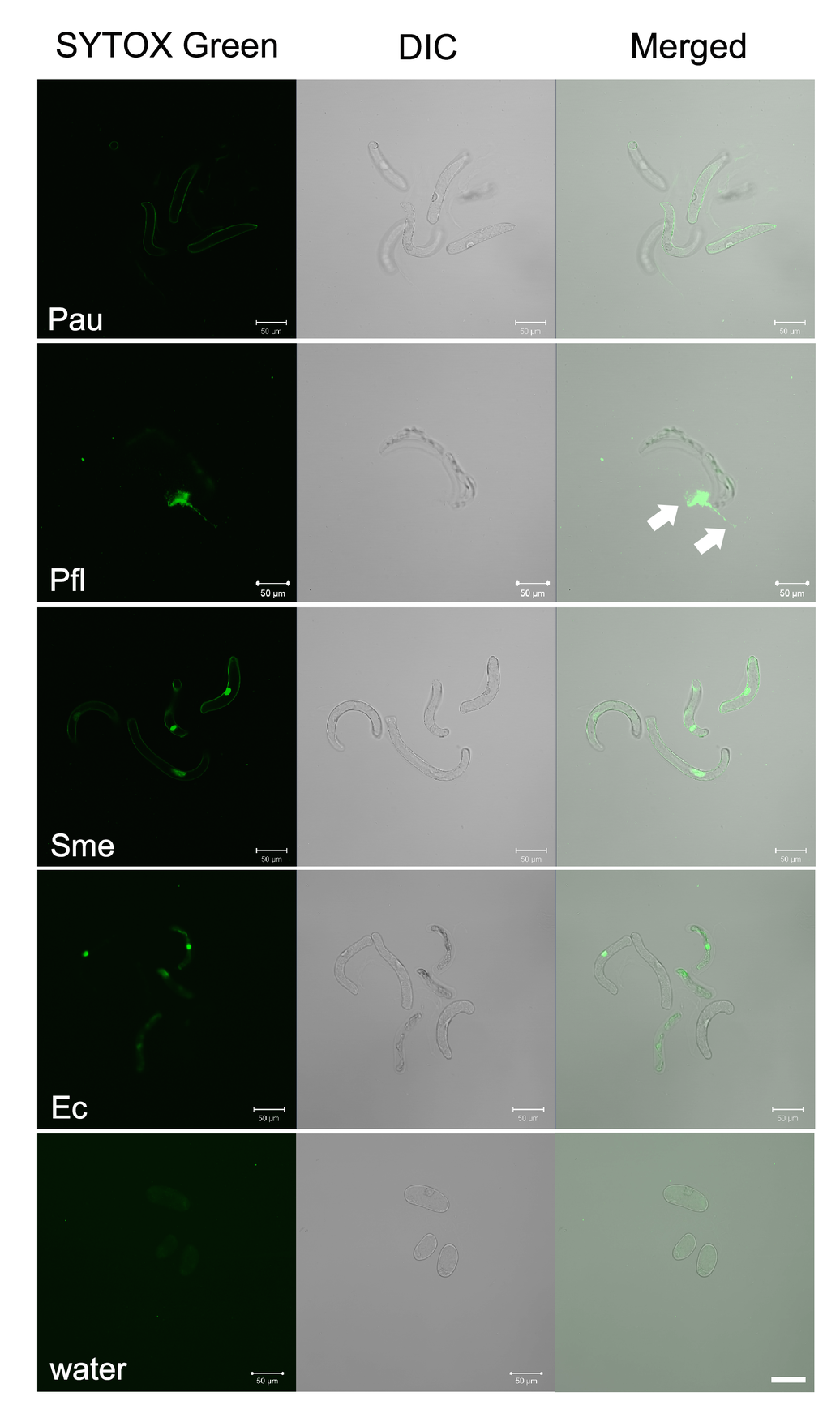

Supplement: S2 Fig — Border cells from pea seedling roots were inoculated with 107 cells of Pseudomonas aureofaciens (Pau), Pseudomonas fluorescens (Pfl), Sinorhizobium meliloti (Sme), E.coli (Ec) or sterile water and stained with SYTOX Green to visualize DNA (white arrows). Live imaging was performed with a Zeiss Elyra 780 CLSM. At least 5 images per treatment were taken between 30 min-1 h post inoculation. Images are representative of two independent experiments (bar = 50 μm). (TIF) [file ppat.1005686.s009.tif]

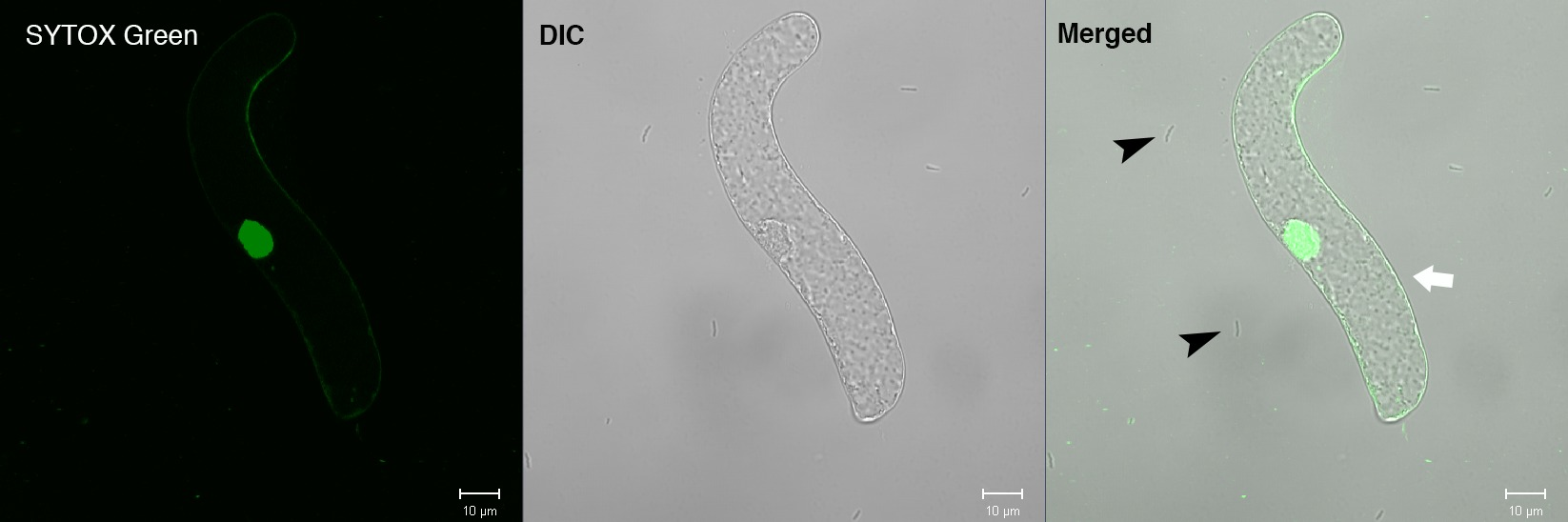

Supplement: S3 Fig — Approximately 10,000 pea border cells were inoculated with 107 CFU of R. solanacearum K60 flagellin mutant fliC. The cell suspension was stained with SYTOX Green and imaged 45 min post inoculation using a Zeiss Elyra 780 CLSM. Extracellular DNA was not observed even when bacteria were close to border cells (arrow heads: bacterial cells; white arrow: pea root border cell). The experiment was repeated three times with similar results. (TIF) [file ppat.1005686.s010.tif]

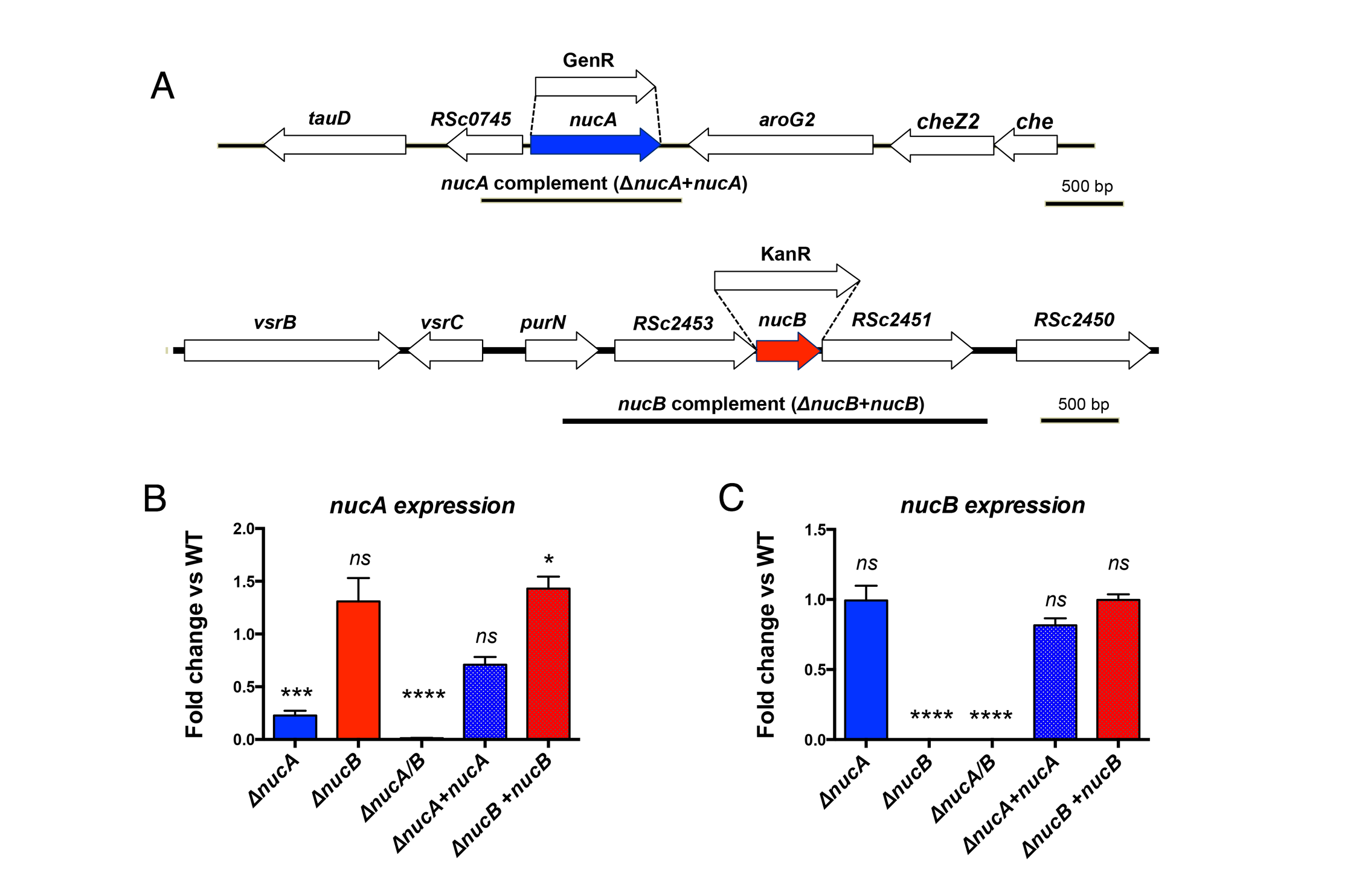

Supplement: S4 Fig — (A) Map showing the genomic context of two putative extracellular nuclease genes in R. solanacearum strain GMI1000 and the location of the antibiotic resistance gene cassettes that replaced the nucA and nucB open reading frames. Arrows indicate open reading frames. (B) and (C) Expression of nucA and nucB in nuclease mutants and complemented mutant strains (ΔnucA+nucA and ΔnucB+nucB), relative to gene expression levels in wild-type GMI1000. RNA was extracted from bacteria cultured in rich CPG medium to 6x10^8^ CFU/ml. Relative transcript abundance was measured using quantitative RT-PCR and normalized to the reference gene rplM. The means of three independent experiments are presented; bars show standard error of the mean. Asterisks indicate differences from wild-type gene expression (one-way ANOVA, ns: P>0.05, * P≤0.05, *** P≤0.001, **** P≤0.0001). (TIF) [file ppat.1005686.s011.tif]

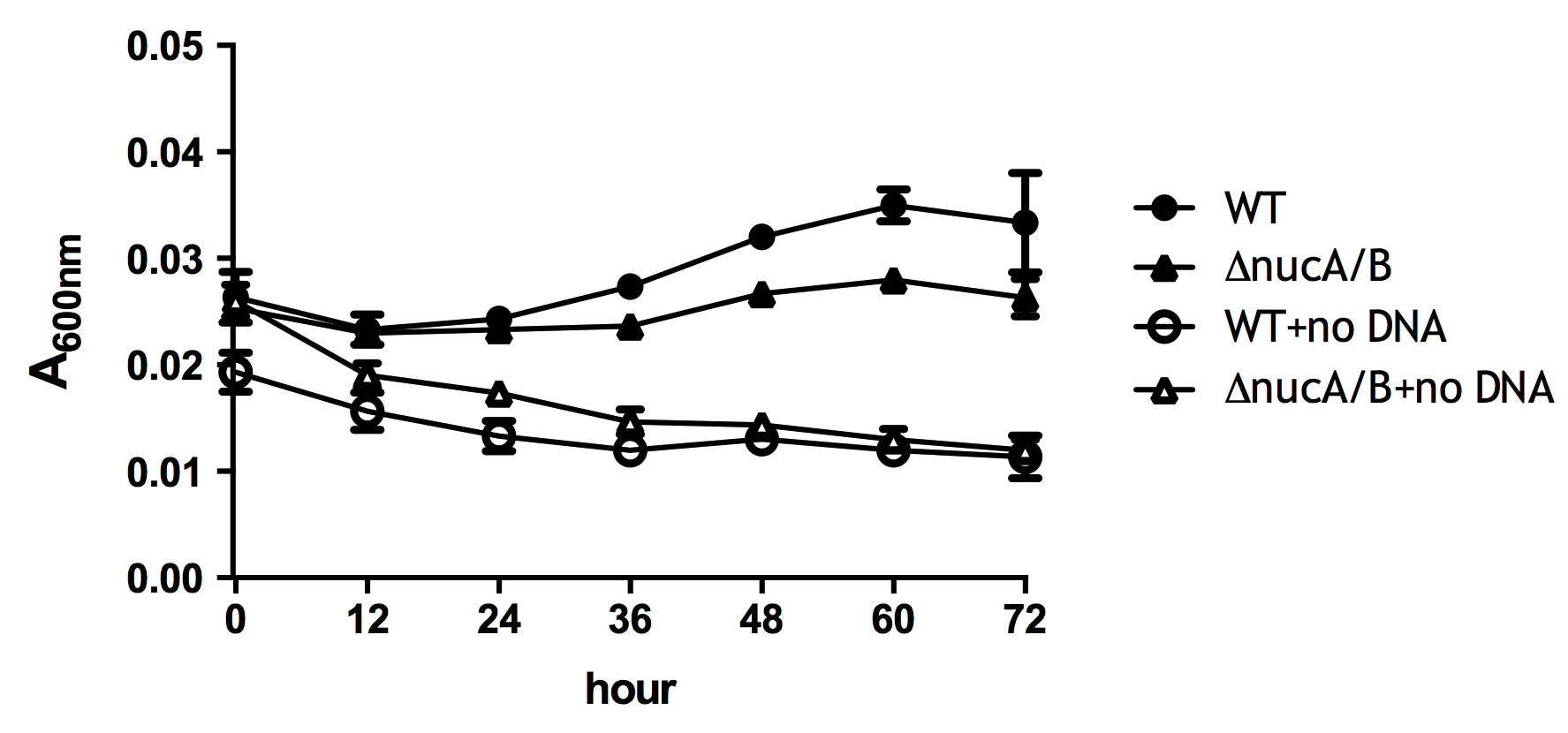

Supplement: S5 Fig — Wild-type R. solanacearum strain GMI1000 and the ΔnucA/nucB double nuclease mutant were grown in minimal medium with or without 5 μg/ml salmon sperm DNA as the sole carbon source. Bacterial growth was measured as absorbance at 600nm using a BioTek plate reader. Strains and growth conditions are indicated as follows: wild-type + DNA, closed circle; ΔnucA/B + DNA, closed triangle; wild-type + no DNA, open circle; ΔnucA/B + no DNA, open triangle (p<0.005, repeated measures ANOVA). (TIF) [file ppat.1005686.s012.tif]

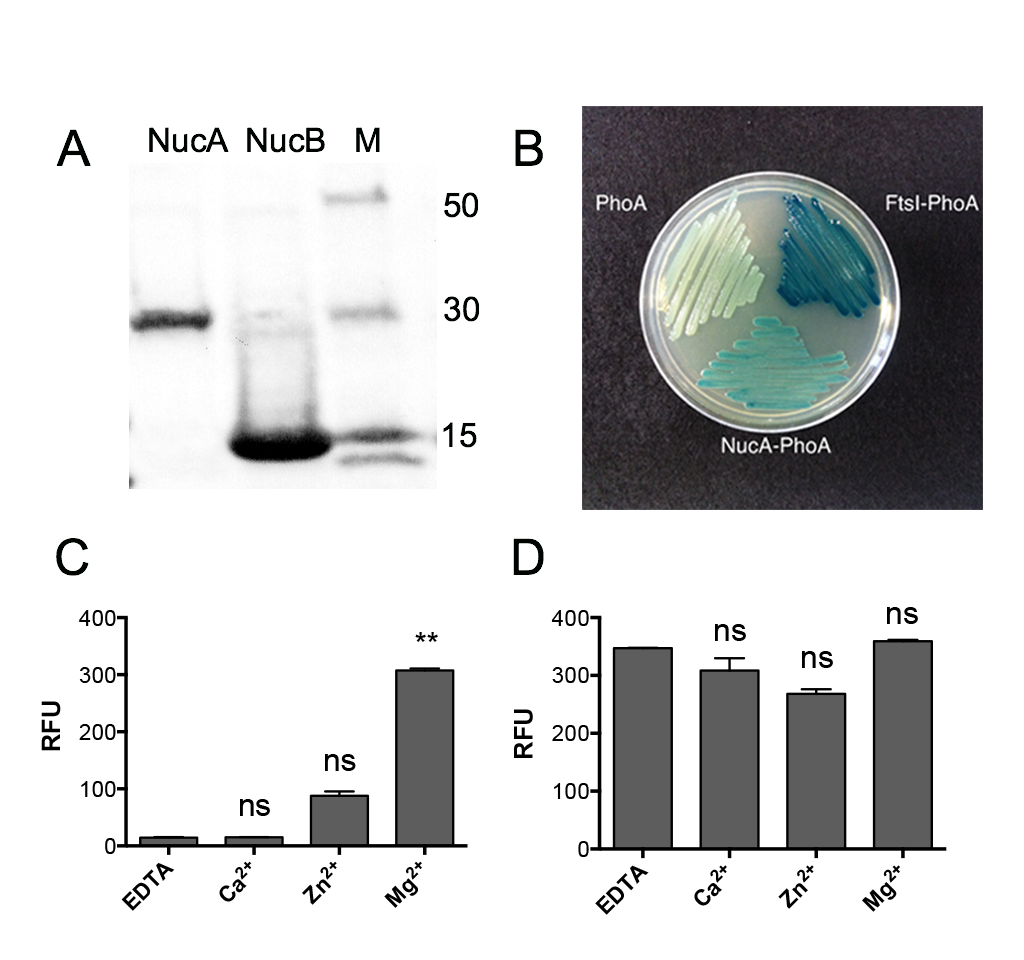

Supplement: S6 Fig — (A) Overexpression plasmid pET29b containing either the nucA or the nucB ORF was transformed into E. coli BL21Star and gene expression was induced with IPTG. The resulting recombinant proteins were purified using nickel columns and detected by Western blot using anti-His antibody (M: 6XHis ladder). (B) Alkaline phosphatase assay of NucA-PhoA fusion in E. coli phoA - strain KS272. Blue or white color of the colonies indicates the PhoA domain is facing the periplasm or the cytoplasm, respectively. (C) and (D) DNase activity of purified NucA (C) and NucB (D) in the presence of different cations or DNase Alert buffer + EDTA. Each reaction contained 2 μg of purified NucA or NucB enzyme. DNase activity was measured by DNase Alert assay using a fluorescence plate reader after 3 h incubation at 37°C. Asterisks indicate difference from EDTA treatment (one-way ANOVA, ** P<0.01). (TIF) [file ppat.1005686.s013.tif]

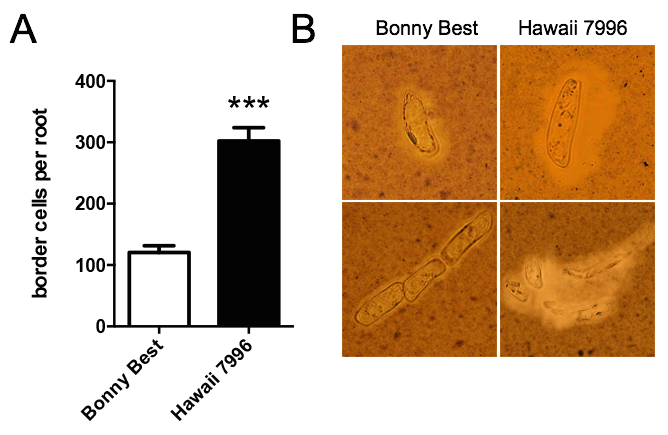

Supplement: S7 Fig — (A) Root border cell counts from 3-day old tomato seedling roots of wilt-susceptible cultivar Bonny Best and wilt-resistant breeding line Hawaii 7996. Seeds from tomato cultivars Hawaii 7996 and Bonny Best were surfaced-sterilized as described above. We germinated the seeds on 1% water agar plates overlaid with filter paper and incubated at 28°C for 4 days. Root border cells were collected by dipping the seedling root tips into sterile water for 1–2 min. The number of border cells was counted under a light microscope as previously described [84]. The experiment was repeated twice, with 10 seedlings for each cultivar. Bars represent manual counts of average border cells from 20 fully germinated seedlings of each cultivar (Student’s t-test, P<0.001). (B) To visualize the slime layer produced by border cells, we collected tomato root border cells from the two cultivars and stained the border cell suspension with India Black ink. Images of both single and clusters of border cells were taken with a light microscope. (TIF) [file ppat.1005686.s014.tif]
